# Peanut shell as a green biomolecule support for anchoring Cu_2_O: a biocatalyst for green synthesis of 1,2,3-triazoles under ultrasonic irradiation

**DOI:** 10.1186/s13065-019-0612-9

**Published:** 2019-07-24

**Authors:** Zahra Dolatkhah, Abolfazl Mohammadkhani, Shahrzad Javanshir, Ayoob Bazgir

**Affiliations:** 1 Heterocyclic Chemistry Research Laboratory, Department of Chemistry, Iran University of Science and Technology, Tehran, 16846-13114 Iran; 2 Department of Chemistry, Shahid Beheshti University, G.C, Tehran, 1983963113 Iran

**Keywords:** Bio-based support, Click reaction, Heterogeneous catalyst, Peanut shell, Triazoles, Ultrasonic irradiation

## Abstract

Cu_2_O supported on peanut shell (Cu_2_O@PS) was prepared by the reaction of copper acetate and peanut shell powder as a naturally available biopolymer support. The prepared catalyst was used as an efficient and reusable heterogeneous catalyst in the click reaction of benzyl halide or phenacyl bromides, acetylenes and sodium azide for the synthesis of potentially biologically active 1,2,3-triazoles under ultrasonic irradiation in EtOH-H_2_O as green solvent.

**Electronic supplementary material:**

The online version of this article (10.1186/s13065-019-0612-9) contains supplementary material, which is available to authorized users.

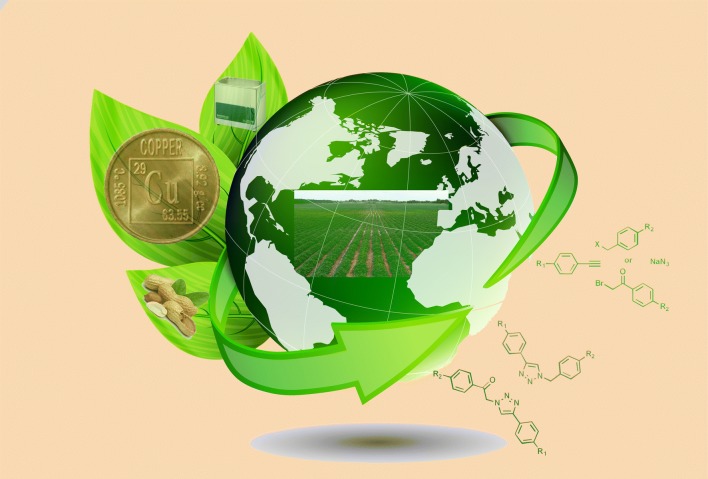

## Introduction

Green chemistry is one of the most important research activities for chemists, both in the laboratory and industry. Therefore, many efficient, eco-friendly and clean synthetic strategies have been developed for the synthesis of biologically and industrially active molecules [1–5]. Meanwhile, metal-catalyzed multi-component reaction is one of the significant areas of green chemistry research. Transition metal-catalyzed click synthesis of triazoles is a powerful method for the synthesis of diverse complex molecules. Triazoles derivatives have developing application in medicinal chemistry and biological activities [6–10]. They also have numerous industrial applications as florescent whiteners, dyestuffs, photo-stabilizers of polymers, and optical brightening agents [11, 12]. Forasmuch as copper-catalyzed click reaction is one of the best methods for the synthesis of 1,2,3-triazoles [13, 14], numerous homogeneous copper catalysts have been reported [15, 16]. Most of these successful methods suffer from non-reusability of the catalysts, and the usage of toxic and/or expensive ligands [17–20]. To overcome these problems, many researchers have focused their efforts on copper-based heterogeneous systems [17, 21, 22].

**Fig. 1:**
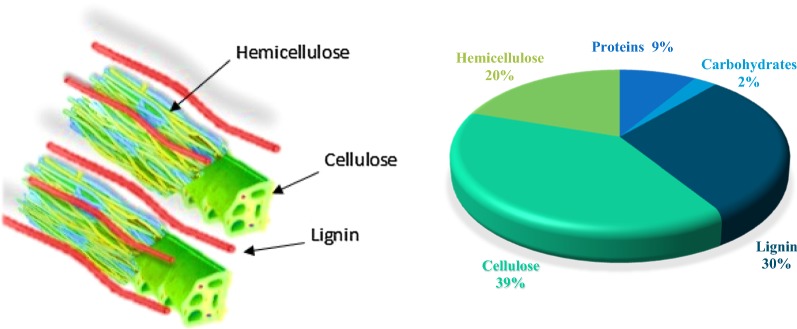
Composition of peanut shell (This figure was designed by authors and it has been taken by authors)

Natural biopolymers are the attractive subjects for the design of bio-supported catalysts due to their eco-friendly, low cost and non-toxic properties [23–30]. Peanut shell as an agro-industrial waste containing considerable fraction of the biodegradable lignocellulosic waste [31] is discarded in the environment or burned about 13.7 million tons per year [32, 33]. This promising natural and renewable raw material consists of a combination of lignin, cellulose, proteins and hemicellulose biopolymers (Fig. 1) [34, 35]. There are many polar functional groups such as hydroxyl, methoxy and carboxyl groups on the surface of peanut shell. Therefore, peanut shell is an attractive candidate as a natural, renewable, non-toxic and very low, or no cost environmentally friendly support for metal nanoparticles.

These days, the application of ultrasonic technology has reported for organic compounds synthesis, emulsification, extraction, nanoparticle formation, and degassing [36–39]. Sonication method has important advantages such as high efficiency, selectivity and yield, economic performance, short reaction time, and low environmental pollution [20, 40–42].

Herein, in continuation of our research toward the development of nano bio-based catalytic systems [43–45], the synthesis of copper oxide supported on peanut shell (Cu_2_O@PS) as a heterogeneous nano-biocatalyst and its catalytic activity for the click synthesis of triazoles in EtOH-H_2_O as green solvent under ultrasonic irradiation is reported.

## Results and discussion

### Synthesis and characterizations of the catalyst

**Scheme 1:**
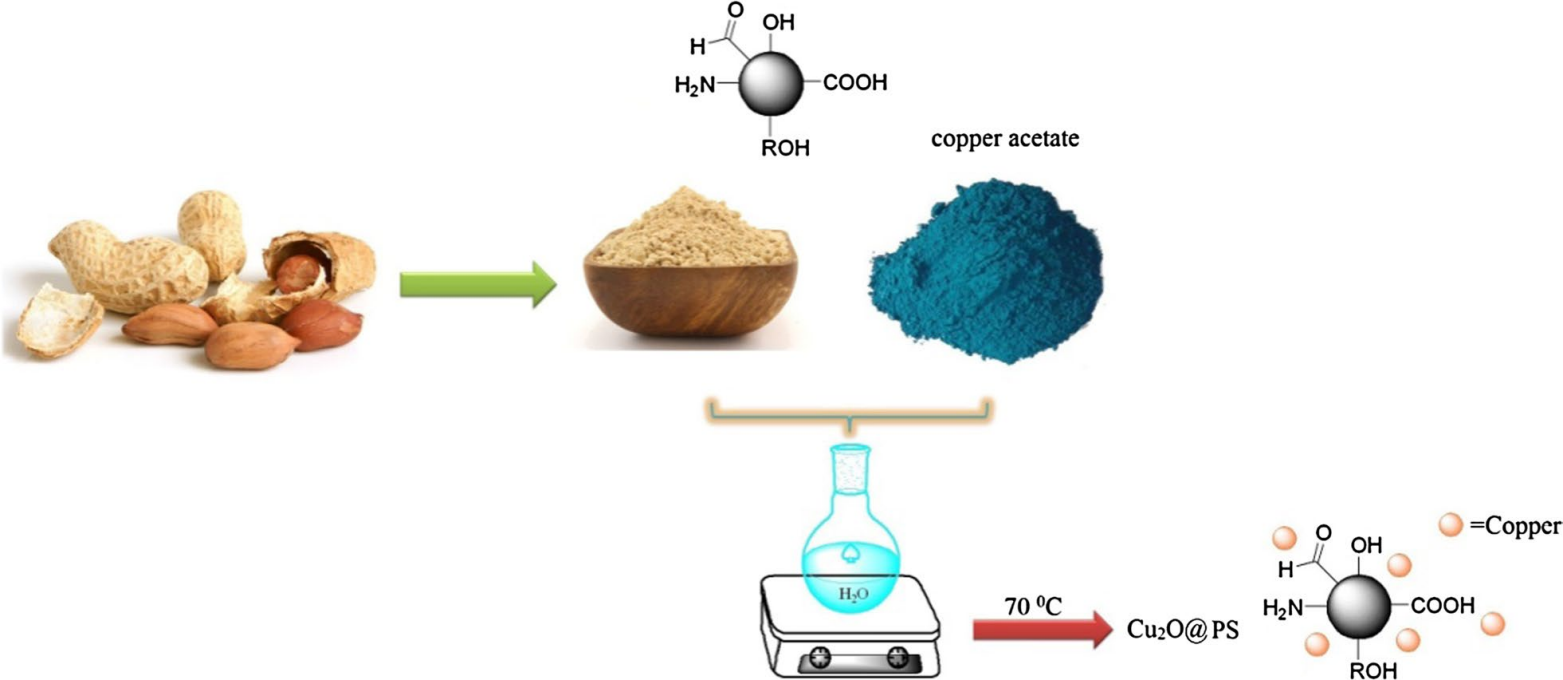
Schematic diagram of catalyst preparation (The source of this diagram is taken from “http://nolinsteel.com/peanuts/” and the used softwares are Chemdraw and Paint. The Scheme was designed by authors)

The preparation of the Cu_2_O@PS nanocomposite is described schematically in Scheme 1. The Cu_2_O@PS nanocomposite obtained by the reaction of peanut shell powder with copper acetate in water at 70 °C for 5 h. The catalyst was centrifuged and washed with water, ethanol, and acetone then dried in the oven at 70 °C.

**Fig. 2:**
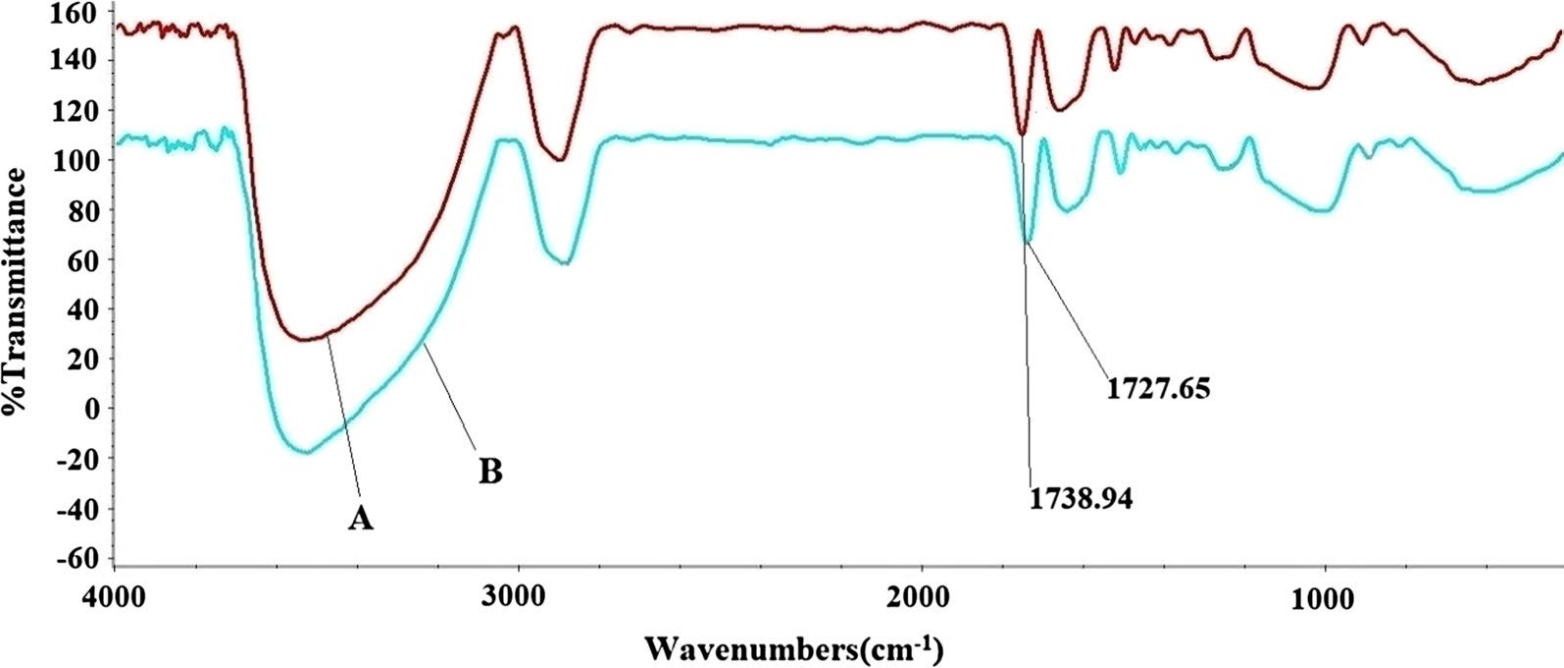
FT-IR spectra of peanut Shell (A), and Cu_2_O@PS (B)

The Cu_2_O@PS nanocomposite was characterized by FT-IR, thermogravimetric analysis (TGA), atomic absorption spectroscopy (AAS), scanning electron microscopy (SEM), energy dispersive X-ray spectroscopy (EDS) analysis and X-ray diffraction (XRD) measurements. The FT-IR spectrum of peanut shell and Cu_2_O@PS are shown in Fig. 2. The band around 3400 cm^−1^ was ascribed to the mixed stretching vibration absorption band of amino and hydroxyl groups. The bands at 2950 cm^−1^ were assigned to aliphatic C–H, mainly CH_2_ stretching. The small band obtained at 1738 cm^−1^ was assigned to the carbonyl groups stretching vibration [29, 46]. As can be seen in the FT-IR spectrum of Cu_2_O@PS, the presence of characteristic bands of PS in the 1738 and 3500 cm^−1^ regions clearly confirms the existence of PS in the final catalyst. Also, the band shift from 1738 cm^−1^ (in PS) to 1727 cm^−1^ (in Cu_2_O@PS) reveals the coordination of copper to peanut shell [46].

**Fig. 3:**
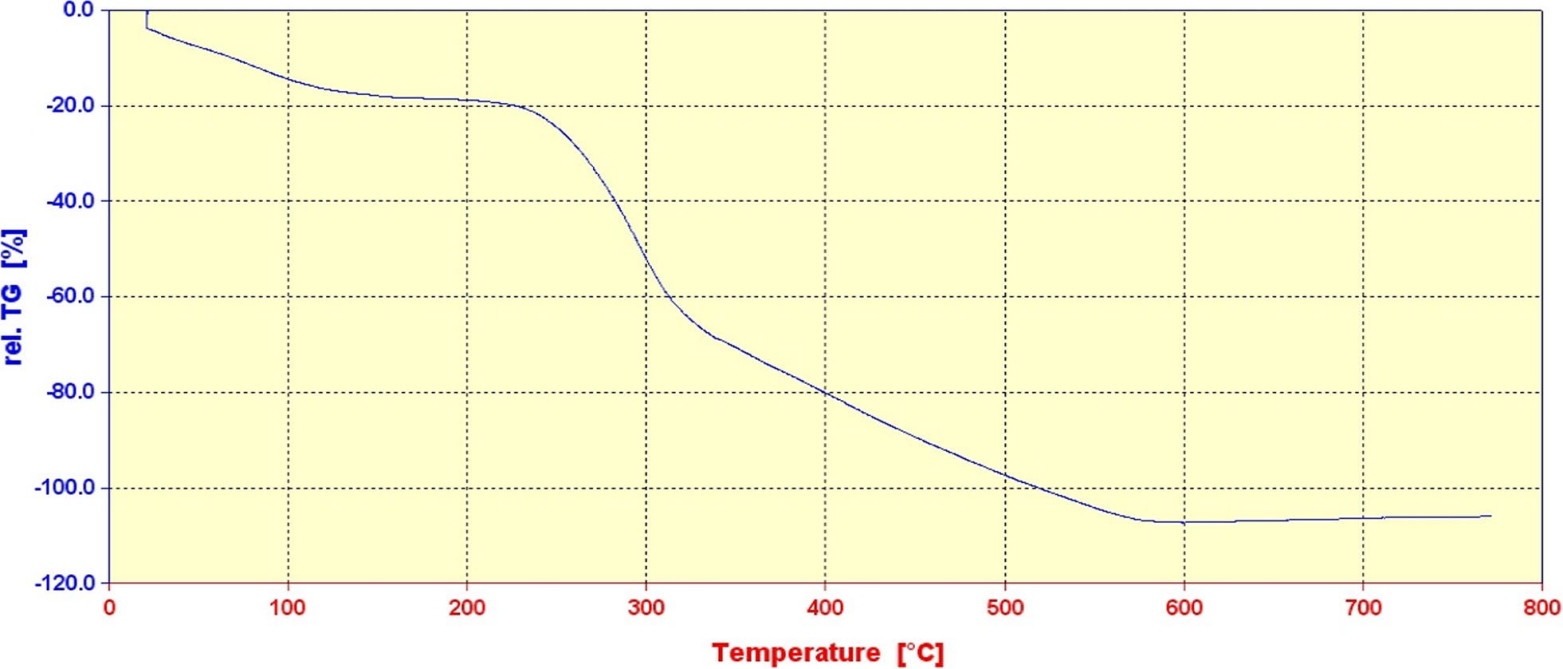
Thermal gravimetric analysis of Cu_2_O@PS

Thermogravimetric analysis was further used to study the composition of the catalyst (Fig. 3). The TGA curve of the catalyst shows a weight loss at ~ 100 °C that is associated with the release of physically adsorbed water. The weight loss above ~ 230 °C (and continued to ~ 600 °C) is related to the decomposition of PS and organic groups on the Cu_2_O@PS. Thermal analysis showed that the catalyst has good thermal stability up to 230 °C. Also, the copper content on the Cu_2_O@PS nanocomposite was measured 0.28 mmol g^−1^ by atomic absorption spectroscopy.

**Fig. 4:**
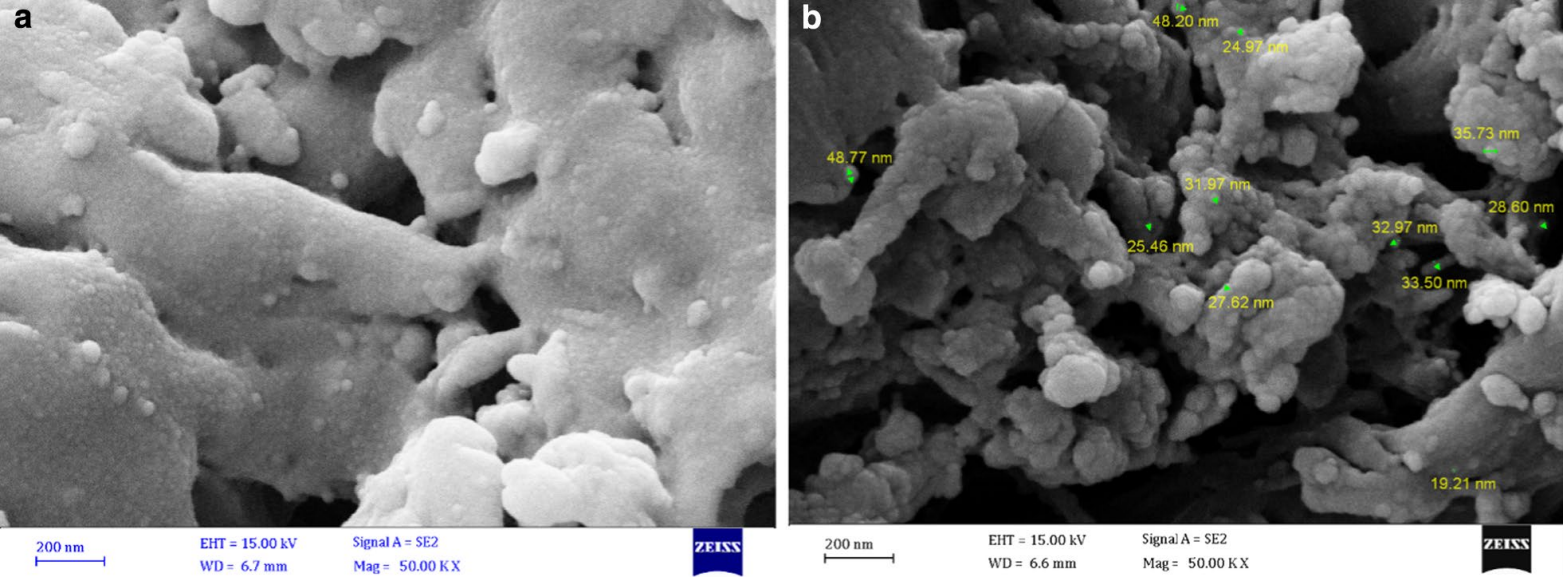
FESEM images of PS (**a**), and Cu_2_O@PS (**b**)

Morphologies of fresh peanut shell and the Cu_2_O@PS nanocomposite were determined by SEM. The fresh PS is basically smooth (Fig. 4a). The SEM images of Cu_2_O@PS show the formation of spherical particles in size around 30–40 nm on the surface of the peanut shell (Fig. 4b).

**Fig. 5:**
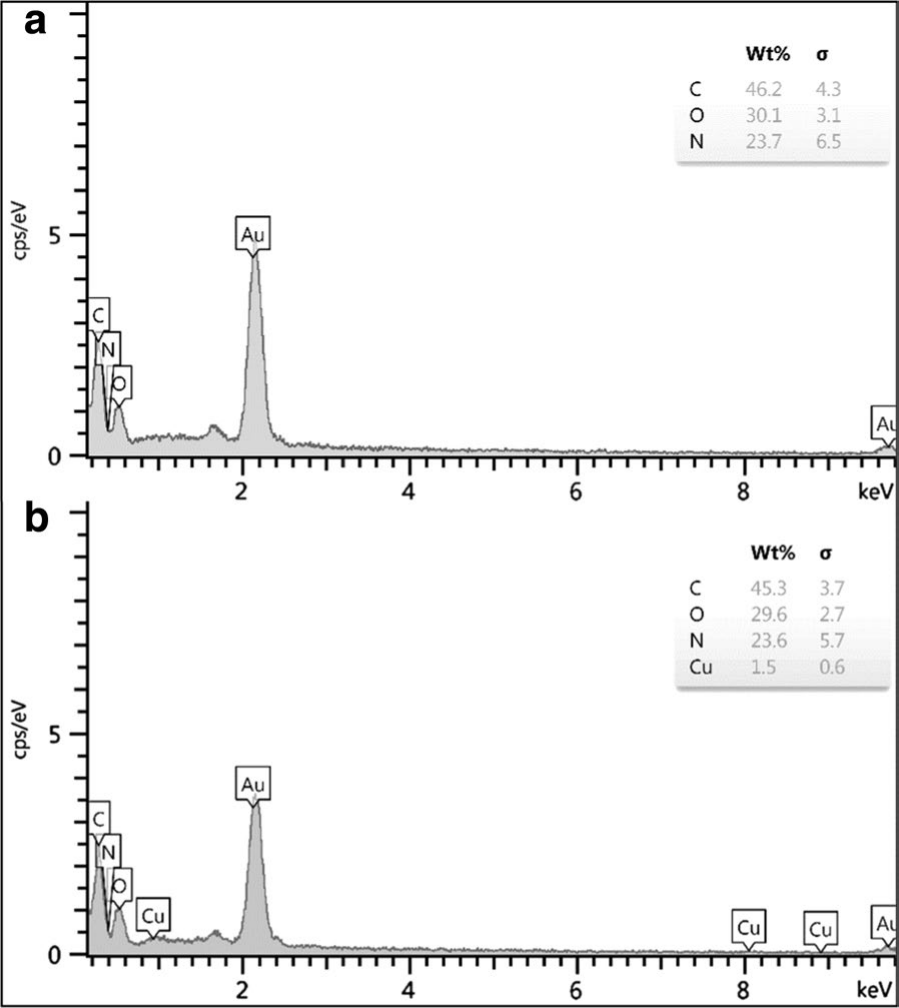
Energy dispersive X-ray spectroscopy (EDS) of PS (**a**), and Cu_2_O@PS (**b**)

**Fig. 6:**
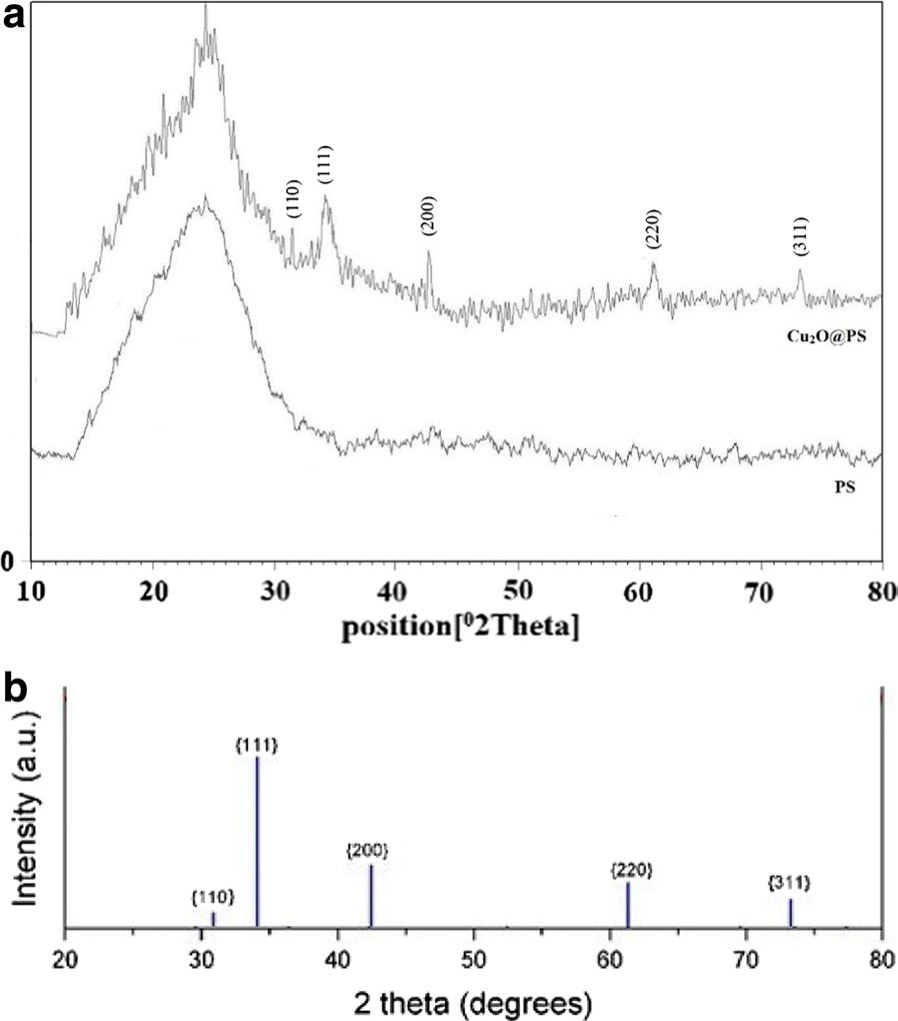
XRD pattern of PS and Cu_2_O@PS nanocomposite (**a**), Cu_2_O (**b**)

In addition, comparing the EDS analysis of Ps and Cu_2_O@PS clearly shows the presence of Cu, C, O, and N elements in this composite and demonstrate that copper was anchored to the PS (Fig. 5a and b). In the XRD pattern of the Cu_2_O@PS nanocomposite, the diffractions at 2θ = 36.4°, 42.5°, 61.4°, and 73.4° can be assigned to the (111), (200), (220) and (311) lattice planes of Cu_2_O, in accordance with Cu_2_O standard data (JCPDS card NO. 05–0667) (Fig. 6).

### Catalytic studies

The catalytic activity of Cu_2_O@PS nanocomposite was investigated in the click synthesis of 1,2,3-triazoles. The reaction of phenyl acetylene **1**, 4-nitrobenzyl bromide **2** and sodium azide (NaN_3_) was chosen as a model reaction under different conditions. As the first experiment, K_2_CO_3_ was used as a base in the presence of 1 mol% of catalyst, and this reaction was tested by employing various solvents such as toluene, MeCN, MeOH, EtOH, H_2_O, EtOH-H_2_O, and MeOH-H_2_O at 50 °C under ultrasonic irradiation for 45 min (Table 1, entries 1–7). A superior yield was obtained when EtOH-H_2_O (1:1) was used as the solvent (Table 1, entry 6). Then, different base were screened in the model reaction. A moderate yields were obtained with Cs_2_CO_3_ and KO*t*-Bu (entries 8 and 9) and the reaction proceed in fairly good yields in the presence of NaOH and KOH (entries 10 and 11). Also, some experiments were carried out at different temperatures, and finally 50 °C was chose as optimum reaction temperature (entries 6, 12–14). Effect of the catalyst loading was also investigated under the optimum reaction conditions. It was found that, when the amount of catalyst increased from 0.5 and 1 to 2 mol%, the yield of product changed from 65 and 91% to 92%, respectively. So, 1 mol% of catalyst is sufficient to promote this reaction (entries 6, 15–16). When this reaction was carried out without catalyst, the yield of the product was trace (entry 17). To delineate the role of ultrasound, the reaction was investigated without ultrasonic irradiation at 50 °C in various solvents. In all reactions, the result obtained by the use of ultrasound irradiation leads to a higher yield (entries 18–20). Finally, when this reaction was carried out with PS or Cu_2_O as catalyst, the yield of the product was trace and 78% yield, respectively (entries 21 and 22).

**Table 1:** Screening of the reaction conditions 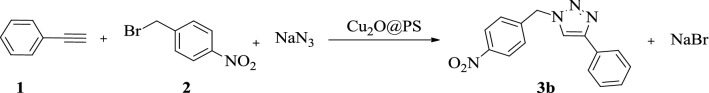

Entry	Solvent/T (°C)	Base	Cat. (mol%)	Method	Yield (%)^a^
1	Toluene/50	K_2_CO_3_	1	Ultrasound	Trace
2	MeCN/50	K_2_CO_3_	1	Ultrasound	65
3	MeOH/50	K_2_CO_3_	1	Ultrasound	77
4	EtOH/50	K_2_CO_3_	1	Ultrasound	72
5	H_2_O/50	K_2_CO_3_	1	Ultrasound	73
6	EtOH-H_2_O/50	K_2_CO_3_	1	Ultrasound	91
7	MeOH-H_2_O/50	K_2_CO_3_	1	Ultrasound	74
8	EtOH-H_2_O/50	Cs_2_CO_3_	1	Ultrasound	53
9	EtOH-H_2_O/50	KO*t*-Bu	1	Ultrasound	49
10	EtOH-H_2_O/50	NaOH	1	Ultrasound	69
11	EtOH-H_2_O/50	KOH	1	Ultrasound	71
12	EtOH-H_2_O/70	K_2_CO_3_	1	Ultrasound	86
13	EtOH-H_2_O/40	K_2_CO_3_	1	Ultrasound	71
14	EtOH-H_2_O/room temperature	K_2_CO_3_	1	Ultrasound	57
15	EtOH-H_2_O/50	K_2_CO_3_	0.5	Ultrasound	65
16	EtOH-H_2_O/50	K_2_CO_3_	2	Ultrasound	92
17	EtOH-H_2_O/50	K_2_CO_3_	–	Ultrasound	Trace
18	EtOH-H_2_O/50	K_2_CO_3_	1	High-speed stirring	56
19	MeOH-H_2_O/50	K_2_CO_3_	1	High-speed stirring	44
20	H_2_O/50	K_2_CO_3_	1	High-speed stirring	48
21^b^	EtOH-H_2_O/50	K_2_CO_3_	40 mg	Ultrasound	Trace
22^c^	EtOH-H_2_O/50	K_2_CO_3_	1	Ultrasound	78

4-Nitro benzyl bromide (1 mmol), phenylacetylene (1.2 mmol), K_2_CO_3_ (2 mmol), NaN_3_ (1.2 mmol), 45 min

^a^Isolated yield

^b^Cat. = PS (40 mg)

^c^Cat. = Cu_2_O (1 mol%)

To explore the scope of the click reaction various benzyl bromides and aryl acetylenes, containing both electron donating and electron withdrawing functionalities were screened in optimized reaction conditions and high isolated yields were obtained (Table 2). Under the same reaction conditions benzyl chlorides provided target products in good yields (Table 2, entries 12–14).

**Table 2:** Click synthesis of 1*H*-1,2,3-triazoles 
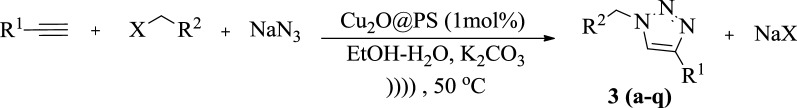

Entry	R^1^	R^2^	X	Product	Yield (%)^a^	M.P. (°C)	Refs.
1	ph	ph	Br	3a	90	131–133	[47]
2	ph	4-NO_2_-ph	Br	3b	91	140–141	[48]
3	4-OMe-ph	ph	Br	3c	86	142–143	[49]
4	4-OMe-ph	4-Me-ph	Br	3d	92	149–151	[50]
5	ph	4-Me-ph	Br	3e	89	106–107	[51]
6	ph	4-Br-ph	Br	3f	93	150–152	[48]
7	4-Me-ph	4-Br-ph	Br	3g	92	202–203	[48]
8	4-OMe-ph	4-NO_2_-ph	Br	3h	97	167–168	[47]
9	4-CF_3_-ph	4-NO_2_-ph	Br	3i	88	215–217	[52]
10	4-Me-ph	ph	Br	3j	90	153–154	[53]
11	4-Me-ph	4-NO_2_-ph	Br	3k	86	159–160	[54]
12	ph	4-OMe-ph	Cl	3l	88	136–138	[49]
13	4-Me-ph	4-OMe-ph	Cl	3m	89	133–136	[51]
14	4-OMe-ph	ph	Cl	3c	88	142–143	[49]
15	ph	*n*-C_3_H_7_	Br	3n	64	41–42	[55]
16	ph	*n*-C_4_H_9_	Br	3o	65	42	[56]
17	CO_2_Me	ph	Br	3p	63	104–105	[57]
18	CO_2_Me	4-NO_2_-ph	Br	3q	61	189–190	[57]

Reaction conditions: benzyl halide (1 mmol), arylacetylene (1.2 mmol), K_2_CO_3_ (2 mmol), NaN_3_ (1.2 mmol), 45 min

^a^Isolated yield

Observation of great potential activity of Cu_2_O@PS nano-biocatalyst in the Click reaction of benzyl bromides and aryl acetylenes encouraged us to investigate the Click reaction of aryl acetylenes **1** with phenacyl bromides **4** and sodium azide in the same reaction conditions. As can be seen from Table 3, the Click reaction of aryl acetylenes and phenacyl bromides contain electron withdrawing or donation groups provide 1*H*-1, 2, 3-triazol-ethan-1-one derivatives **5** in good isolated yields in the presence of 1 mol% of catalyst in EtOH-H_2_O under ultrasound irradiation at 50 °C.

**Table 3:** Synthesis of 1*H*-1, 2, 3-triazol-ethan-1-one derivatives by Click reaction 
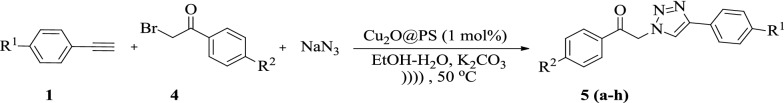

Entry	R^1^	R^2^	Product	Yield (%)^a^	M.P. (°C)	Refs.
1	H	H	5a	93	166–167	[58]
2	OMe	H	5b	89	190–191	[58]
3	H	Br	5c	86	145–146	[59]
4	H	NO_2_	5d	77	180–181	[60]
5	H	Cl	5e	89	150–152	[59]
6	H	OMe	5f	88	142–143	[59]
7	CF_3_	H	5g	81	221–223	[58]
8	Me	H	5h	85	165–167	[58]

Phenacyl bromides (1 mmol), arylacetylene (1.2 mmol), K_2_CO_3_ (2 mmol), NaN_3_ (1.2 mmol), Cu_2_O@PS (1 mol%), 90 min

^a^Isolated yield

**Fig. 7:**
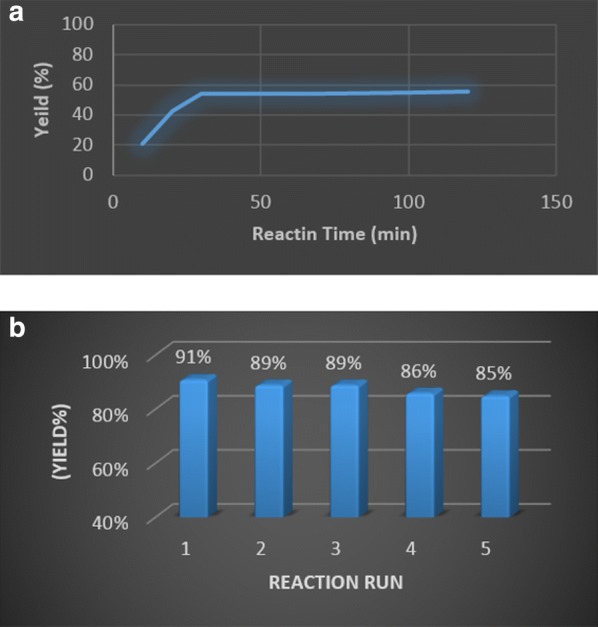
Hot filtration test observed by GC analysis (**a**), the reusability of the catalyst (**b**)

Then, we examined the heterogeneous nature of the catalyst. Firstly, to assess the copper leaching of the catalyst, we performed hot filtration test for the click reaction of 4-nitro benzyl bromide **2**, phenylacetylene **1** and NaN_3_. The reaction was stopped after ~ 50% of the reaction time. Hot filtrate was transferred to another flask containing base and H_2_O-EtOH at 50 °C. Upon further heating of the catalyst-free solution for 1.5 h, no considerable progress was observed by GC analysis (Fig. 7a). Moreover, atomic absorption spectroscopy (AAS) of the same reaction solution at the midpoint of completion indicated that no significant quantities of copper were lost to the reaction medium during the process. Furthermore, the reusability of catalyst was investigated in the reaction of 4-nitro benzyl bromide, phenylacetylene, and NaN_3_. The catalyst could be reused successively five times without significant loss of activity (Fig. 7b). Moreover, atomic absorption spectroscopy revealed that the loading of copper was 0.27 mmol g^−1^ after five runs and there was no significant change in the copper content of the recovered catalyst. All results confirm the reaction occurs mainly via a heterogeneous pathway. The SEM micrographs of reused catalyst after five times reveal that the reused catalyst has a similar texture with fresh catalyst (see Additional file 1).

**Scheme 2:**
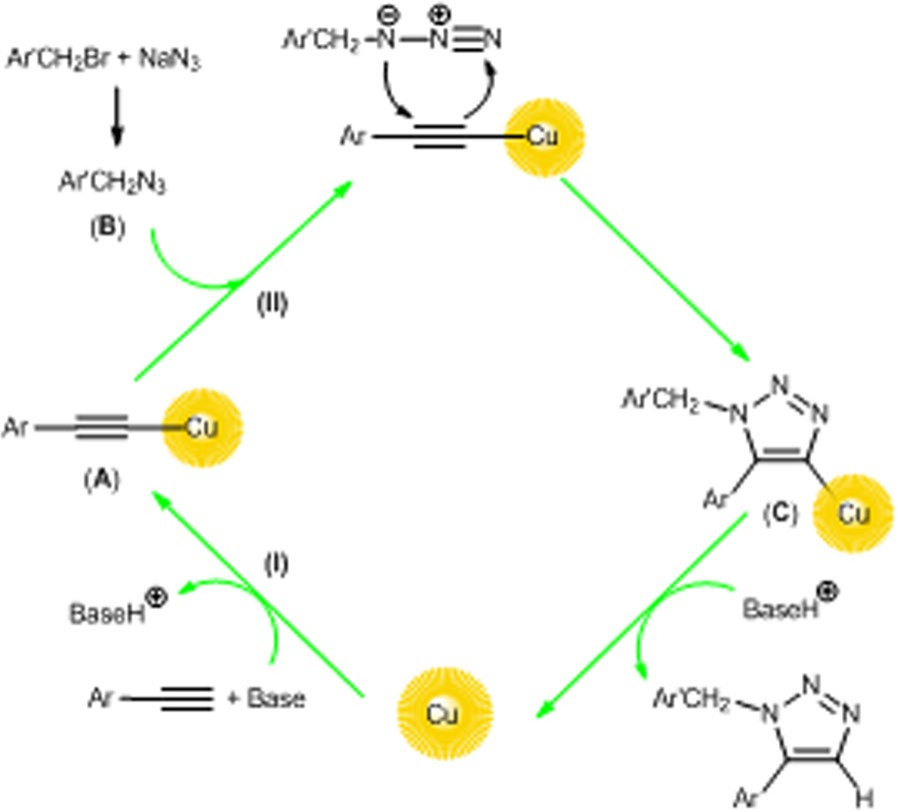
The proposed mechanism of the reaction. The Scheme was designed by authors (The sources of Graphical abstract are internet “https://pngtree.com/freepng/vector-arrow-earth_520723.html”, “http://aisphysicalscience.pbworks.com/w/page/1623001/29%20Cu%20-%20Copper” and “https://pngtree.com/so/peanut-shells” and the used softwares are Chemdraw and Paint. The Scheme was designed by authors)

Based on literature reports [61], a possible mechanism for click catalytic synthesis of triazole is proposed in Scheme 2. Synthesis of triazole proceeds through the formation of copper acetylide (A). The coordination of organic azide (B) (formed in situ by the reaction of organic bromide with NaN_3_) to the copper acetylide, followed by the Huisgen 1, 3-dipolar cycloaddition reaction of (A) and (B) give the complex (C). Subsequently, the desired triazole was obtained by copper-acidic hydrogen exchange followed by regeneration of the catalyst for the next use in the catalytic cycle. It is notable; functional groups such as hydroxyl, amine, methoxy and carboxyl groups on the surface of peanut shell have good potential to coordinate with copper nanoparticles.

Reports in Table 4, compares the efficiency of Cu_2_O@PS nanocomposite with some other heterogeneous copper catalysts in literature in the Click reaction of phenylacetylene, phenacyl bromides and NaN_3_. Table 4 shows that although all of methods have good efficiency, the present catalyst affords some advantages such as biodegradability using green nano bio-support for immobilization of copper, reasonable reaction time and low temperature which are all energy and time-consuming processes.

**Table 4:** Comparison of efficiency of various heterogeneous catalysts for triazole synthesis

Catalyst	Cu (mmol g^−1^)	Catalyst (mol%)	Time (h)	T (°C)	Solvent	Yield (%)	Refs.
GO/Pim/Cu	2.1	1	1.5	50	H_2_O	90	[62]
Cell-CuI NPs	0.37	3.7	2	70	H_2_O	96	[51]
P[imCl/IL][Cu]	1.3	0.1	3.5	55	H_2_O/tBuOH	98	[63]
Mag-Cu	0.46	2	6	55	H_2_O/tBuOH	93	[64]
PANI@CuI-NPs	0.94	5	0.5	100	H_2_O	85	[65]
Fe_3_O_4_@SiO_2_-ABT/Cu(OAc)_2_	3	0.02	2	70	PEG/H_2_O	85	[66]
Cu_2_O-Ag NPs	2.8	3.5	2	25	H_2_O-EtOH	92	[67]
Cu_2_O@PS	0.28	1	1.5	50	H_2_O-EtOH	93	This work

## Experimental

### Material and measurements

All chemicals were purchased from Merck, Aldrich or Fluka were used without further purification. IR spectra were recorded on a Shimadzu FT-IR-470 FT-IR spectrophotometer. EDS characterization was performed using an electron microscopy Oxford Instrument Company, Germany. Field emission scanning electron microscopy (FESEM) was performed using a ZEISS instrument, SIGMA VP model, Germany. The NMR spectra were recorded on a Brukerdrx-300Avance spectrometer. The concentration of Cu was estimated using a Shimadzu AA-680 flame atomic absorption spectrophotometer. Diffraction data were collected on a STOE STADI P with scintillation detector, secondary monochromator and Cu-Ka1 radiation (λ = 1. 5406 Å). Gas chromatography was performed on a Trace GC ultra from the Thermo Company equipped with FID detector and Rtx^®^-1 capillary column. Melting points of products were measured with an Electrothermal 9100 apparatus and are uncorrected. Thermogravimetric analysis (TGA) was done by D-32609 Hullhorst. The peanut shell was obtained from Astaneh Ashrafiyeh Township located in 37° 16′ latitude and 49° 56′ longitude in north of Iran.

### Preparation of Cu_2_O@Peanut shell

Crushed peanut shells were ground in a ball mill to a fine powder. A mixture of peanut shell powder (1 g) and copper acetate (0.1 g) was stirred in de-ionized water (30 ml) at 70 °C for 5 h. The catalyst was then centrifuged and washed with water, ethanol, and acetone and dried in the oven at 70 °C to obtain Cu_2_O@Peanut shell.

### General procedure for Click reactions

A mixture of Cu_2_O@PS (1 mol% of Cu, 40 mg), K_2_CO_3_ (2 mmol), aryl bromide (1.0 mmol), phenyl acetylene (1.2 mmol), and NaN_3_ (1.2 mmol) in H_2_O-EtOH (3 ml, 1:1) was sonicated at 50 °C for an appropriate time. After completion of the reaction monitored by TLC (EtOAc:*n*-hexane (1:3), the catalyst was separated and the filtrate was extracted with Chloroform (2 × 2 ml). The organic solvents were removed under vacuum and the pure product was obtained by recrystallization with CHCl_3_:*n*-hexane (1:3). All of the Click products are known compound and were reported previously.

## Conclusions

In summary, Cu_2_O@Peanut shell nano-biocomposite was synthesized and used as an effective heterogeneous catalyst in a one-pot Huisgen 1,3-dipolar cycloaddition reaction under ultrasonic irradiation in EtOH-H_2_O as a green solvent for the synthesis of 1,2,3-triazole derivatives. The reusability of the catalyst is high and the catalyst can be reused five times without a significant decrease in its catalytic activity. Notable features of this catalytic reaction are bio-degradable and bio-renewable polymeric support, compatibility with a wide range of substrate, mild reaction conditions, high atom economy, good-yields of the products, ligand-free, leaching-free and eco-friendliness characteristics of the catalyst.

## Additional file

**Additional file 1.** Supporting information including the FESEM images of PS, Cu_2_O@PS, and reused Cu_2_O@PS after 5 times, characterization of triazole products, and HNMR spectrum of products.

## Data Availability

All data generated or analysed during this study are included in this published article [and its Additional file 1].

## References

[1] Bai L, Wang J-X, Zhang Y (2003). Rapid microwave-promoted Suzuki cross coupling reaction in water. Green Chem.

[2] Pironti V, Colonna S (2005). Microwave-promoted synthesis of [small beta]-hydroxy sulfides and [small beta]-hydroxy sulfoxides in water. Green Chem.

[3] Leadbeater NE (2005). Fast, easy, clean chemistry by using water as a solvent and microwave heating: the Suzuki coupling as an illustration. Chem Commun.

[4] Al-Amin M, Akimoto M, Tameno T, Ohki Y, Takahashi N, Hoshiya N (2013). Suzuki-Miyaura cross-coupling reactions using a low-leaching and highly recyclable gold-supported palladium material and two types of microwave equipments. Green Chem.

[5] Frost CG, Mutton L (2010). Heterogeneous catalytic synthesis using microreactor technology. Green Chem.

[6] Klich K, Pyta K, Kubicka MM, Ruszkowski P, Celewicz L, Gajecka M (2016). Synthesis, antibacterial, and anticancer evaluation of novel spiramycin-like conjugates containing C(5) triazole arm. J Med Chem.

[7] Velázquez S, Alvarez R, Pérez C, Gago F, Clercq ED, Balzarini J (1998). Regiospecific synthesis and anti-human immunodeficiency virus activity of novel 5-substituted *N*-alkylcarbamoyl and *N*, *N*-dialkyl carbamoyl 1,2,3-triazole-TSAO analogues. Antivir Chem Chemother.

[8] Pereira D, Fernandes P (2011). Synthesis and antibacterial activity of novel 4-aryl-[1,2,3]-triazole containing macrolides. Bioorg Med Chem Lett.

[9] Buckle DR, Outred DJ, Rockell CJM, Smith H, Spicer BA (1983). Studies on v-triazoles. 7. Antiallergic 9-oxo-1*H*,9*H*-benzopyrano[2,3-d]-v-triazoles. J Med Chem.

[10] Fung-Tomc JC, Huczko E, Minassian B, Bonner DP (1998). In vitro activity of a new oral triazole, BMS-207147 (ER-30346). Antimicrob Agents Chemother.

[11] Wamhoff H (1984). Comprehensive heterocyclic chemistry.

[12] Fan W-Q, Katritzsky AR (1996). Comprehensive heterocyclic chemistry II.

[13] Aucagne V, Leigh DA (2006). Chemoselective formation of successive triazole linkages in One Pot: “Click–Click” chemistry. Org Lett.

[14] Siemsen P, Livingston RC, Diederich F (2000). Acetylenic coupling: a powerful tool in molecular construction. Angew Chem Int Ed.

[15] Astruc D, Liang L, Rapakousiou A, Ruiz J (2012). Click dendrimers and triazole-related aspects: catalysts, mechanism, synthesis, and functions. A bridge between dendritic architectures and nanomaterials. Acc Chem Res.

[16] Gawande MB, Bonifacio VDB, Luque R, Branco PS, Varma RS (2013). Benign by design: catalyst-free in-water, on-water green chemical methodologies in organic synthesis. Chem Soc Rev.

[17] Lal K, Rani P. Recent developments in copper nanoparticle-catalyzed synthesis of 1,4-disubstituted 1,2,3-triazoles in water. Arkivoc. 2016;i:307–341

[18] Huisgen R (1989). Kinetics and reaction mechanisms: selected examples from the experience of forty years. Pure Appl Chem.

[19] Pressly ED, Amir RJ, Hawker CJ (2011). Rapid synthesis of block and cyclic copolymers via click chemistry in the presence of copper nanoparticles. J Polym Sci Part A Polym Chem.

[20] Cintas P, Barge A, Tagliapietra S, Boffa L, Cravotto G (2010). Alkyne–azide click reaction catalyzed by metallic copper under ultrasound. Nat Protoc.

[21] Ghosh S, Saha S, Sengupta D, Chattopadhyay S, De G, Basu B (2017). Stabilized Cu_2_O nanoparticles on macroporous polystyrene resins [Cu_2_O@ARF]: improved and reusable heterogeneous catalyst for on-water synthesis of triazoles via click reaction. Ind Eng Chem Res.

[22] Khodaei MM, Bahrami K, Meibodi FS (2017). Ferromagnetic nanoparticle-supported copper complex: a highly efficient and reusable catalyst for three-component syntheses of 1, 4-disubstituted 1, 2, 3-triazoles and C-S coupling of aryl halides. Appl Organomet Chem.

[23] Zhou P, Wang H, Yang J, Tang J, Sun D, Tang W (2012). Bacteria cellulose nanofibers supported palladium (0) nanocomposite and its catalysis evaluation in heck reaction. Ind Eng Chem Res.

[24] Rezaei R, Sheikhi MR (2015). Starch–sulfuric acid as a bio-supported and recyclable solid acid catalyst for rapid synthesis of α, α′-benzylidene bis(4-hydroxycoumarin) derivatives. Res Chem Intermed.

[25] Sin E, Yi S-S, Lee Y-S (2010). Chitosan-g-mPEG-supported palladium (0) catalyst for Suzuki cross-coupling reaction in water. J Mol Catal A Chem.

[26] Mohammad Zaheri H, Javanshir S, Hemmati B, Dolatkhah Z, Fardpour M (2018). Magnetic core–shell Carrageenan moss/Fe_3_O_4_: a polysaccharide-based metallic nanoparticles for synthesis of pyrimidinone derivatives via Biginelli reaction. Chem Cent J.

[27] Punnadiyil RK, Sreejith MP, Purushothaman E (2016). Isolation of microcrystalline and nano cellulose from peanut shells. J Chem Pharm Sci.

[28] Bao C, Ma J, Zhou L, Shao Y, Wu Q, Wang F (2015). Self-template synthesis of hierarchical magnetic porous carbon fibers derived from Fe(BTC)-coated bamboo fibers for fast removal of methylene blue. RSC Adv.

[29] Zhu C-S, Wang L-P, Chen W-B (2009). Removal of Cu(II) from aqueous solution by agricultural by-product: peanut hull. J Hazard Mater.

[30] Johnson P, Watson M, Brown J, Jefcoat I (2002). Peanut hull pellets as a single use sorbent for the capture of Cu(II) from wastewater. Waste Manag.

[31] Kumar M, Revathi K, Khanna S (2015). Biodegradation of cellulosic and lignocellulosic waste by *Pseudoxanthomonas* sp R-28. Carbohydr Polym.

[32] Zhao X, Chen J, Du F (2012). Potential use of peanut by-products in food processing: a review. J Food Sci Technol.

[33] Ding J, Wang H, Li Z, Cui K, Karpuzov D, Tan X (2015). Peanut shell hybrid sodium ion capacitor with extreme energy-power rivals lithium ion capacitors. Energy Environ Sci.

[34] Anike FN, Yusuf M, Isikhuemhen OS (2016). Co-substrating of peanut shells with cornstalks enhances biodegradation by *Pleurotus ostreatus*. J Bioremediat Biodegrad.

[35] Tanyildizi MŞ (2011). Modeling of adsorption isotherms and kinetics of reactive dye from aqueous solution by peanut hull. Chem Eng J.

[36] Serra S, Fuganti C, Brenna E (2005). Biocatalytic preparation of natural flavours and fragrances. Trends Biotechnol.

[37] Azizi N, Gholibeglo E, Maryami M, Nayeri SD, Bolourtchian SM (2013). Ultrasound mediated efficient ring opening of epoxides by in situ generated dithiocarbamates in green reaction media. C R Chim.

[38] Chen G-F, Jia H-M, Zhang L-Y, Chen B-H, Li J-T (2013). An efficient synthesis of 2-substituted benzothiazoles in the presence of FeCl_3_/Montmorillonite K-10 under ultrasound irradiation. Ultrason Sonochem.

[39] Zhang Z, Zha Z, Gan C, Pan C, Zhou Y, Wang Z (2006). Catalysis and regioselectivity of the aqueous Heck reaction by Pd(0) nanoparticles under ultrasonic irradiation. J Org Chem.

[40] Kaur G, Sharma A, Banerjee B (2018). Ultrasound and ionic liquid: an ideal combination for organic transformations. ChemistrySelect.

[41] Banerjee B (2017). Recent developments on ultrasound assisted catalyst-free organic synthesis. Ultrason Sonochem.

[42] Tasdelen MA, Kiskan B, Yagci Y (2016). Externally stimulated click reactions for macromolecular syntheses. Prog Polym Sci.

[43] Hemmati B, Javanshir S, Dolatkhah Z (2016). Hybrid magnetic Irish moss/Fe_3_O_4_ as a nano-biocatalyst for synthesis of imidazopyrimidine derivatives. RSC Adv.

[44] Javanshir S, Saghiran Pourshiri N, Dolatkhah Z, Farhadnia M (2017). Caspian Isinglass, a versatile and sustainable biocatalyst for domino synthesis of spirooxindoles and spiroacenaphthylenes in water. Monatsh Chem.

[45] Pourian E, Javanshir S, Dolatkhah Z, Molaei S, Maleki A (2018). Ultrasonic-assisted preparation, characterization, and use of novel biocompatible core/shell Fe_3_O_4_@GA@ isinglass in the synthesis of 1, 4-dihydropyridine and 4*H*-pyran derivatives. ACS Omega.

[46] Liu S, Xu W-H, Liu Y-G, Tan X-F, Zeng G-M, Li X (2017). Facile synthesis of Cu(II) impregnated biochar with enhanced adsorption activity for the removal of doxycycline hydrochloride from water. Sci Total Environ.

[47] Reddy VH, Reddy YVR, Sridhar B, Reddy BVS (2016). Green catalytic process for click synthesis promoted by copper oxide nanocomposite supported on graphene oxide. Adv Synth Catal.

[48] Naeimi H, Shaabani R (2017). Ultrasound promoted facile one pot synthesis of triazole derivatives catalyzed by functionalized graphene oxide Cu(I) complex under mild conditions. Ultrason Sonochem.

[49] Asano K, Matsubara S (2010). Effects of a flexible alkyl chain on a ligand for CuAAC reaction. Org Lett.

[50] Szadkowska A, Staszko S, Zaorska E, Pawlowski R (2016). A theophylline based copper *N*-heterocyclic carbene complex: synthesis and activity studies in green media. RSC Adv.

[51] Chavan PV, Pandit KS, Desai UV, Kulkarni MA, Wadgaonkar PP (2014). Cellulose supported cuprous iodide nanoparticles (Cell-CuI NPs): a new heterogeneous and recyclable catalyst for the one pot synthesis of 1,4-disubstituted-1,2,3-triazoles in water. RSC Adv.

[52] Shaabani A, Afshari R, Hooshmand SE, Tabatabaei AT, Hajishaabanha F (2016). Copper supported on MWCNT-guanidine acetic acid@Fe_3_O_4_: synthesis, characterization and application as a novel multi-task nanocatalyst for preparation of triazoles and bis(indolyl)methanes in water. RSC Adv.

[53] Kalhor-Monfared S, Beauvineau C, Scherman D, Girard C (2016). Synthesis and cytotoxicity evaluation of aryl triazolic derivatives and their hydroxymethine homologues against B16 melanoma cell line. Eur J Med Chem.

[54] Yamada YMA, Sarkar SM, Uozumi Y (2012). Amphiphilic self-assembled polymeric copper catalyst to parts per million levels: click chemistry. J Am Chem Soc.

[55] Pourjavadi A, Tajbakhsh M, Farhang M, Hosseini SH (2015). Copper-loaded polymeric magnetic nanocatalysts as retrievable and robust heterogeneous catalysts for click reactions. New J Chem.

[56] Dige NC, Patil JD, Pore DM (2017). Dicationic 1, 3-Bis (1-methyl-1*H*-imidazol-3-ium) propane copper(I) dibromate: novel heterogeneous catalyst for 1, 3-dipolar cycloaddition. Catal Lett.

[57] Movassagh B, Rezaei N (2014). Polystyrene resin-supported CuI-cryptand 22 complex: a highly efficient and reusable catalyst for three-component synthesis of 1, 4-disubstituted 1, 2, 3-triazoles under aerobic conditions in water. Tetrahedron.

[58] Cha H, Lee K, Chi DY (2017). Synthesis of N-unsubstituted 1,2,3-triazoles via aerobic oxidative *N*-dealkylation using copper(II) acetate. Tetrahedron.

[59] Ahmady AZ, Heidarizadeh F, Keshavarz M (2013). Ionic liquid containing copper(I): a new, green, homogeneous, and reusable catalyst for click cyclization. Synth Commun.

[60] Gaikwad S, Goswami A, De S, Schmittel M (2016). A metalloregulated four-state nanoswitch controls two-step sequential catalysis in an eleven-component system. Angew Chem Int Ed.

[61] Tajbakhsh M, Farhang M, Baghbanian SM, Hosseinzadeh R, Tajbakhsh M (2015). Nano magnetite supported metal ions as robust, efficient and recyclable catalysts for green synthesis of propargylamines and 1,4-disubstituted 1,2,3-triazoles in water. New J Chem.

[62] Pourjavadi A, Safaie N, Hosseini SH, Bennett C (2015). Graphene oxide/poly (vinyl imidazole) nanocomposite: an effective support for preparation of highly loaded heterogeneous copper catalyst. Appl Organomet Chem.

[63] Pourjavadi A, Hosseini SH, Moghaddam FM, Ayati SE (2015). Copper loaded cross-linked poly (ionic liquid): robust heterogeneous catalyst in ppm amount. RSC Adv.

[64] Banan A, Bayat A, Valizadeh H (2017). Copper immobilized onto polymer-coated magnetic nanoparticles as recoverable catalyst for ‘click’reaction. Appl Organomet Chem.

[65] Saadat S, Nazari S, Afshari M, Shahabi M, Keshavarz M (2015). Copper (I) iodide nanoparticles on polyaniline as a green, recoverable and reusable catalyst for multicomponent click synthesis of 1, 4-disubstituted-1H-1, 2, 3-triazoles. Orient J Chem.

[66] Jafari AA, Mahmoudi H, Firouzabadi H (2015). A copper acetate/2-aminobenzenthiol complex supported on magnetite/silica nanoparticles as a highly active and recyclable catalyst for 1, 2, 3-triazole synthesis. RSC Adv.

[67] Singh G, Kumar M, Bhalla V (2018). Supramolecular ensemble of perylene bisimide derivative and Cu_2_O-Ag nanoparticles: nano/“Dip Strip” catalytic system for one-pot, three-component click reaction at room temperature. ACS Sustain Chem Eng.

