# Supplementary material for: Peanut shell as a green biomolecule support for anchoring Cu2O: a biocatalyst for green synthesis of 1,2,3-triazoles under ultrasonic irradiation
Source: BMC Chem. 2019 Jul 24;13(1):97. doi: 10.1186/s13065-019-0612-9 (PMC6659571; doi:10.1186/s13065-019-0612-9)
Supplement: Supplementary file 1 — Additional file 1. Supporting information including the FESEM images of PS, Cu2O@PS, and reused Cu2O@PS after 5 times, characterization of triazole products, and HNMR spectrum of products. [file 13065_2019_612_MOESM1_ESM.docx]

ADDITIONAL INFORMATION

Peanut shell as a green biomolecule support for anchoring Cu_2_O: A biocatalyst for green synthesis of 1,2,3-triazoles under ultrasonic irradiation

Zahra Dolatkhah^1^, Abolfazl Mohammadkhani^2^, Shahrzad Javanshir^1^, Ayoob Bazgir^2*^**‎**

*^1^Heterocyclic Chemistry Research Laboratory, Department of Chemistry, Iran University of Science and Technology, Tehran 16846-13114, Iran.*

*^2^Department of Chemistry, Shahid Beheshti University, G.C., Tehran 1983963113, Iran*

[*a_bazgir@sbu.ac.ir*](mailto:a_bazgir@sbu.ac.ir)

**Characterization of fresh and reused Cu_2_O@PS catalyst**

**
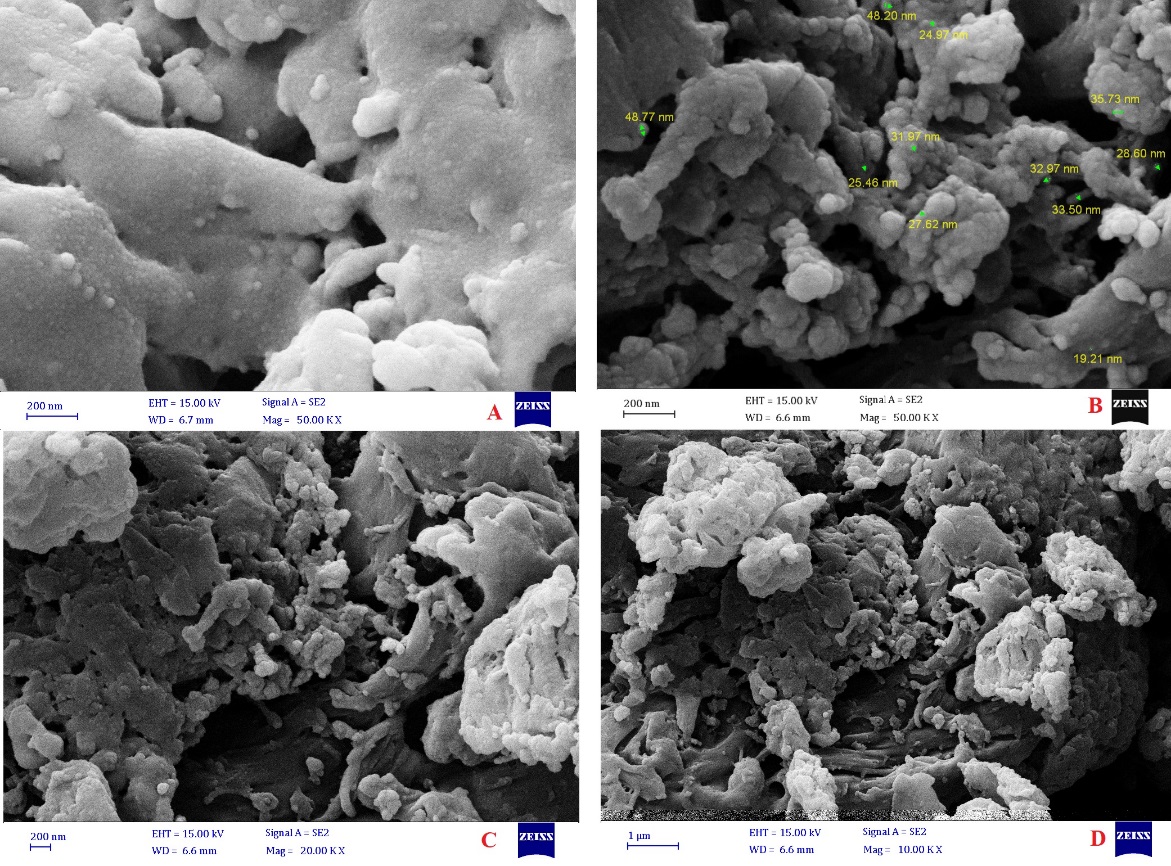
**

**
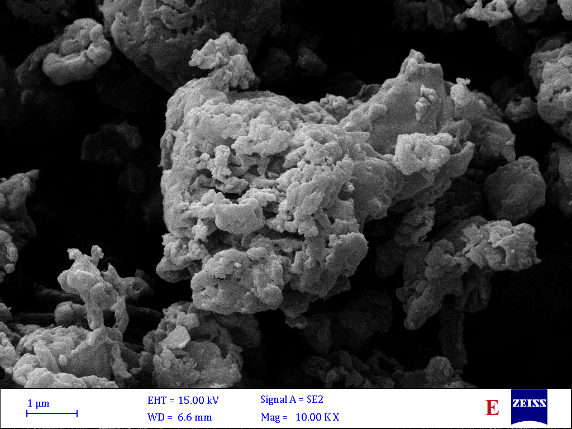

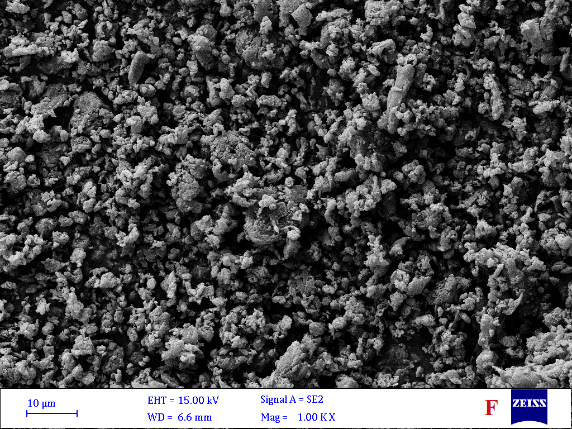
**

**Fig. S1.**FESEM images of PS (A),Cu_2_O@PS (B-D), and reused Cu_2_O@PS after 5 times (E-F)

**Characterization of triazole products**

|  | 1-benzyl-4-phenyl-1*H*-1,2,3-triazole (**3a**): white powder, mp 131-133 ºC^(1)^. ^1^H NMR (300 MHz, CDCl_3_): δ=5.6 (2H, s, CH_2_benzylic), 7.34-7.83 (10H, m, H aromatic), 7.69(1H, s,CHtriazole). |
| --- | --- |
| 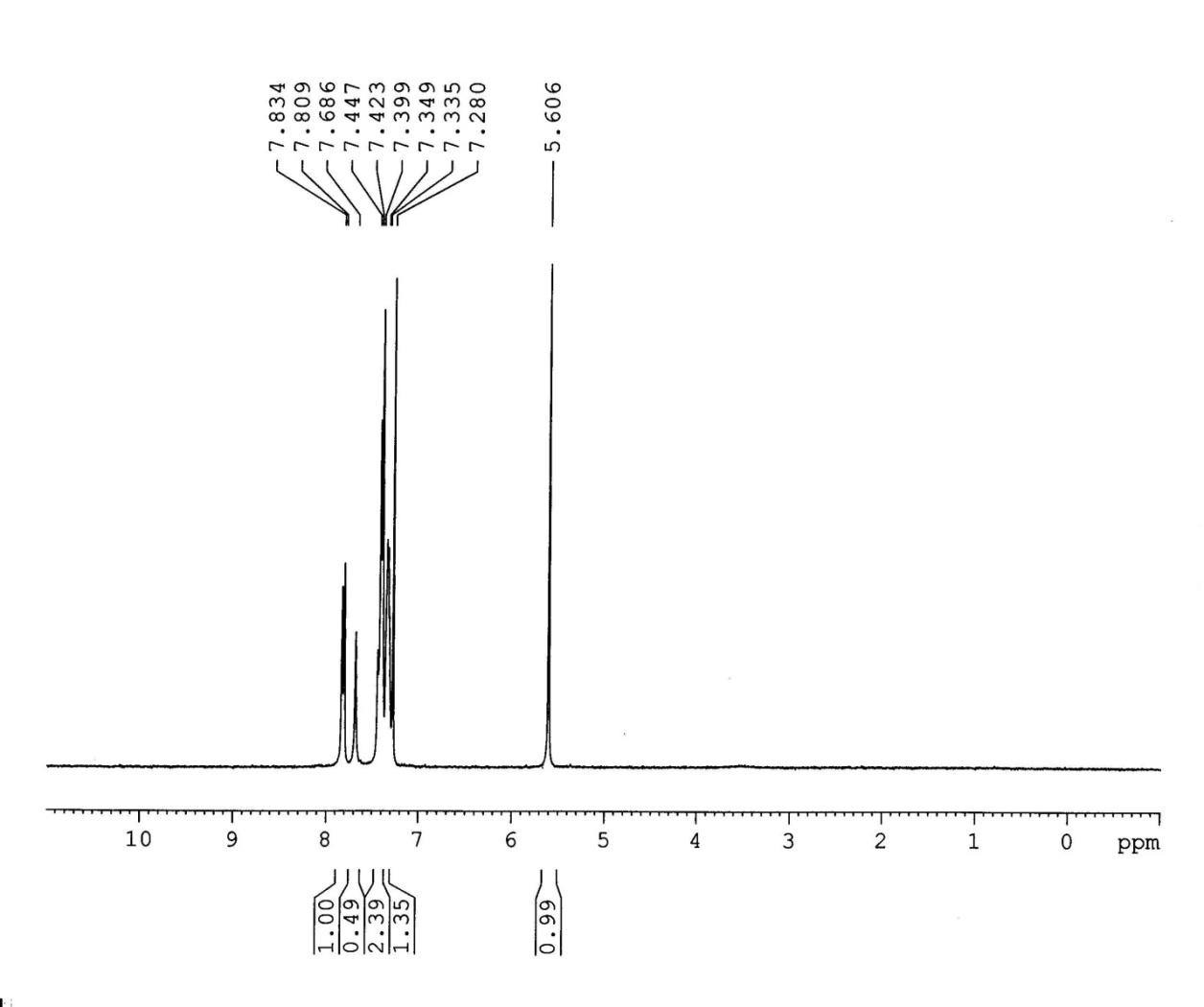 | |

**Fig. S2.**^1^H NMR of **3a** product in CDCl_3_

|  | 1-(4-nitrobenzyl)-4-phenyl-1*H*-1,2,3-triazole (**3b**): yellow powder, mp 140-141ºC ^(2)^.^1^H NMR (300 MHz, CDCl_3_):δ=5.73 (1H ,s, CH_2_benzylic), 7.35-8.29 (9H, m, H aromatic), 7.77(1H, s,CHtriazole). |
| --- | --- |
| 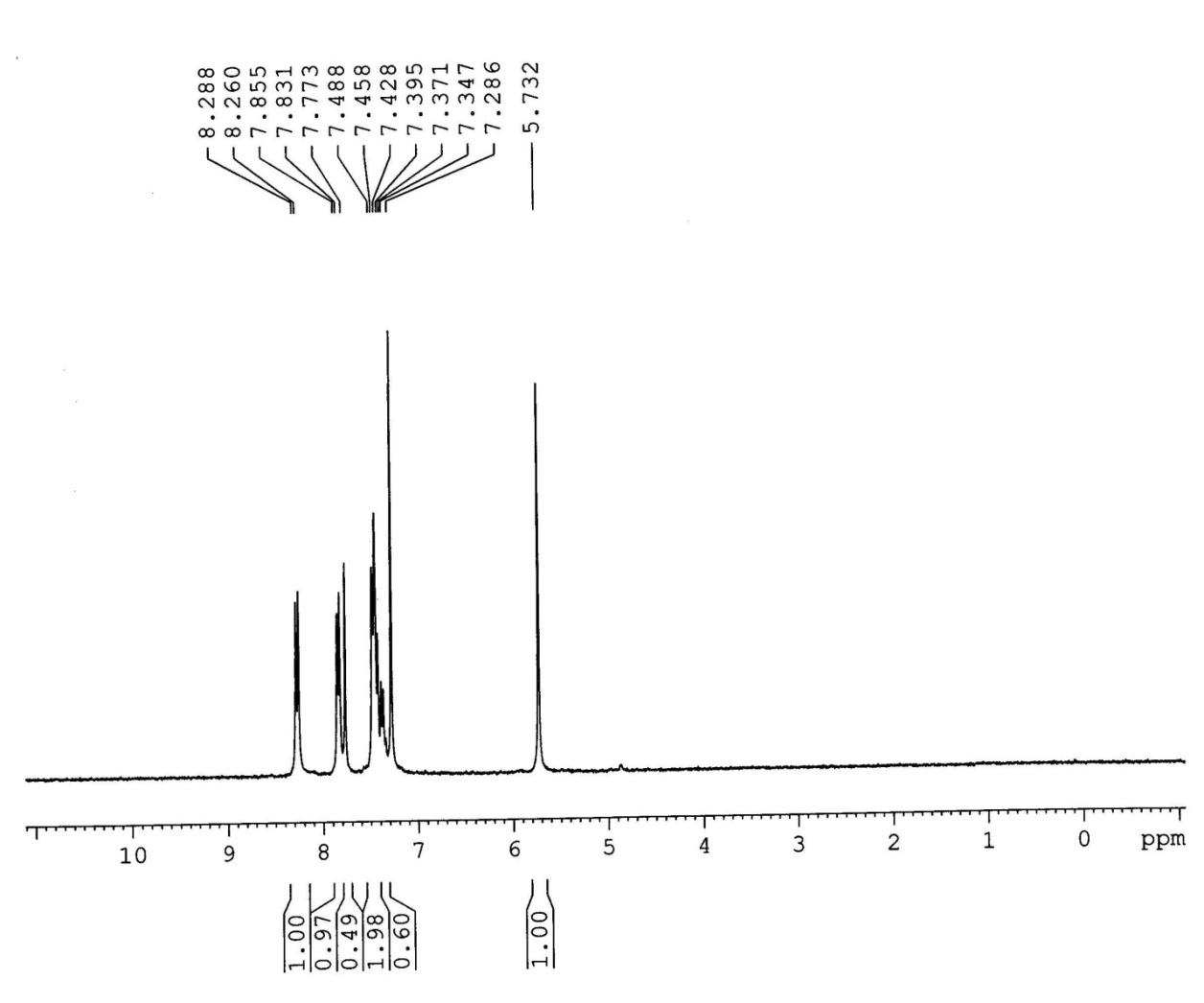 | |

**Fig. S3.**^1^H NMR of **3b** product in CDCl_3_

|  | 1-benzyl-4-(4-methoxyphenyl)-1*H*-1,2,3-triazole (**3c**):white powder, mp 141-142 ºC^(3)^. ^1^H NMR (300 MHz, CDCl_3_): δ=3.86 (3 H, s, OCH_3_), 5.59 (2H, s, CH_2_ benzylic), 6.95-7.76 (9H, m, H aromatic), 7.6 (1H, s, CH triazole). |
| --- | --- |
| 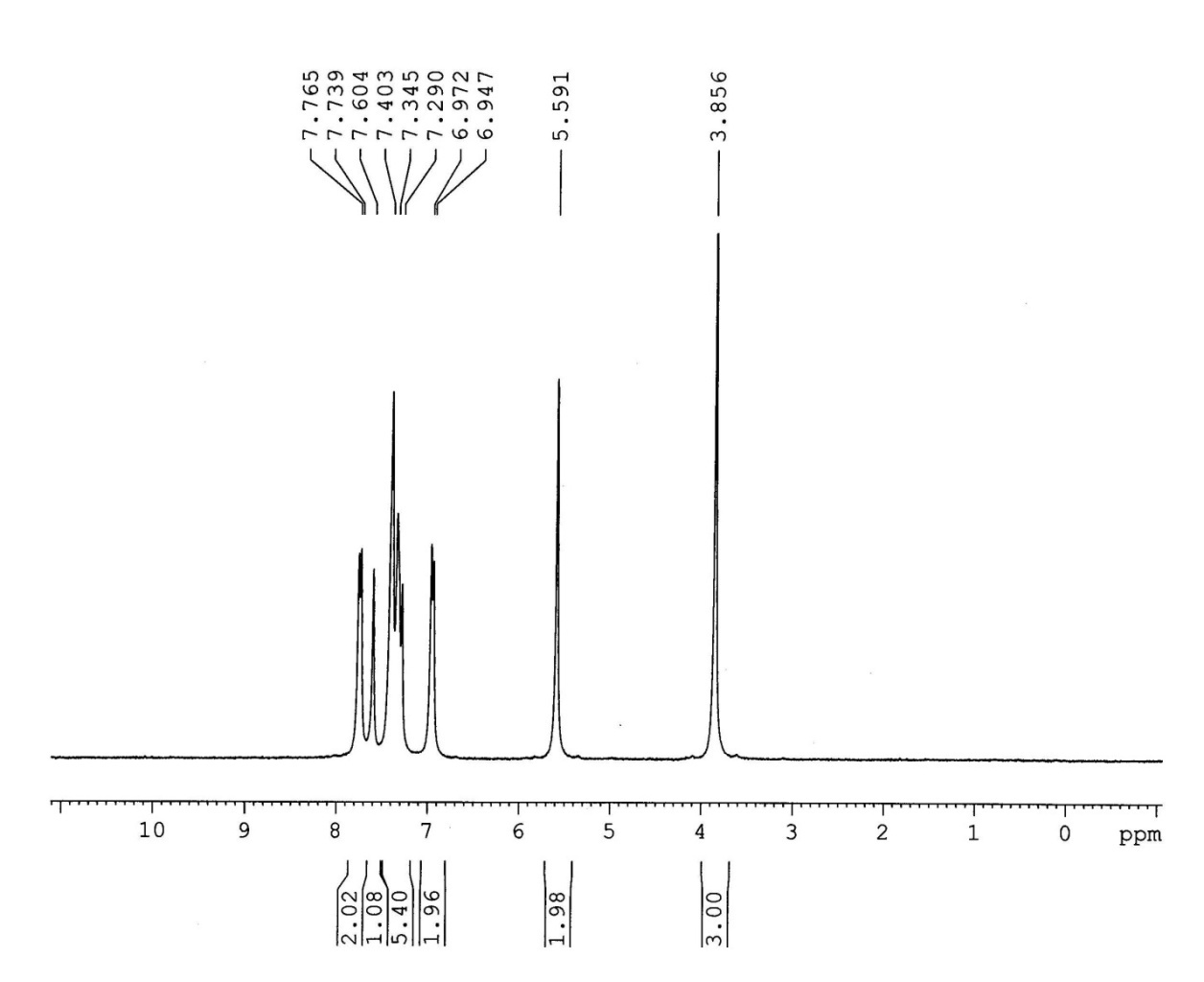 | |

**Fig. S4.**^1^H NMR of **3c** product in CDCl_3_

|  | 4-(4-methoxyphenyl)-1-(4-methylbenzyl)-1*H*-1,2,3-triazole(**3d**): white powder, mp 149-151 ºC ^(4)^.^1^H NMR (300 MHz, CDCl_3_): δ=2.38 (3H ,s, CH_3_) , 3.85 (3H, s, OCH_3_), 5.54 (2H ,s, CH_2_benzylic), 6.94-6.97 (2H, d, J=8.4 Hz, H aromatic), 7.23-7.29 (4H, bs, H aromatic),7.73-7.75 (2H, d, J=8.4 Hz, H aromatic), 7.57 (1H, s,CHtriazole) |
| --- | --- |
| 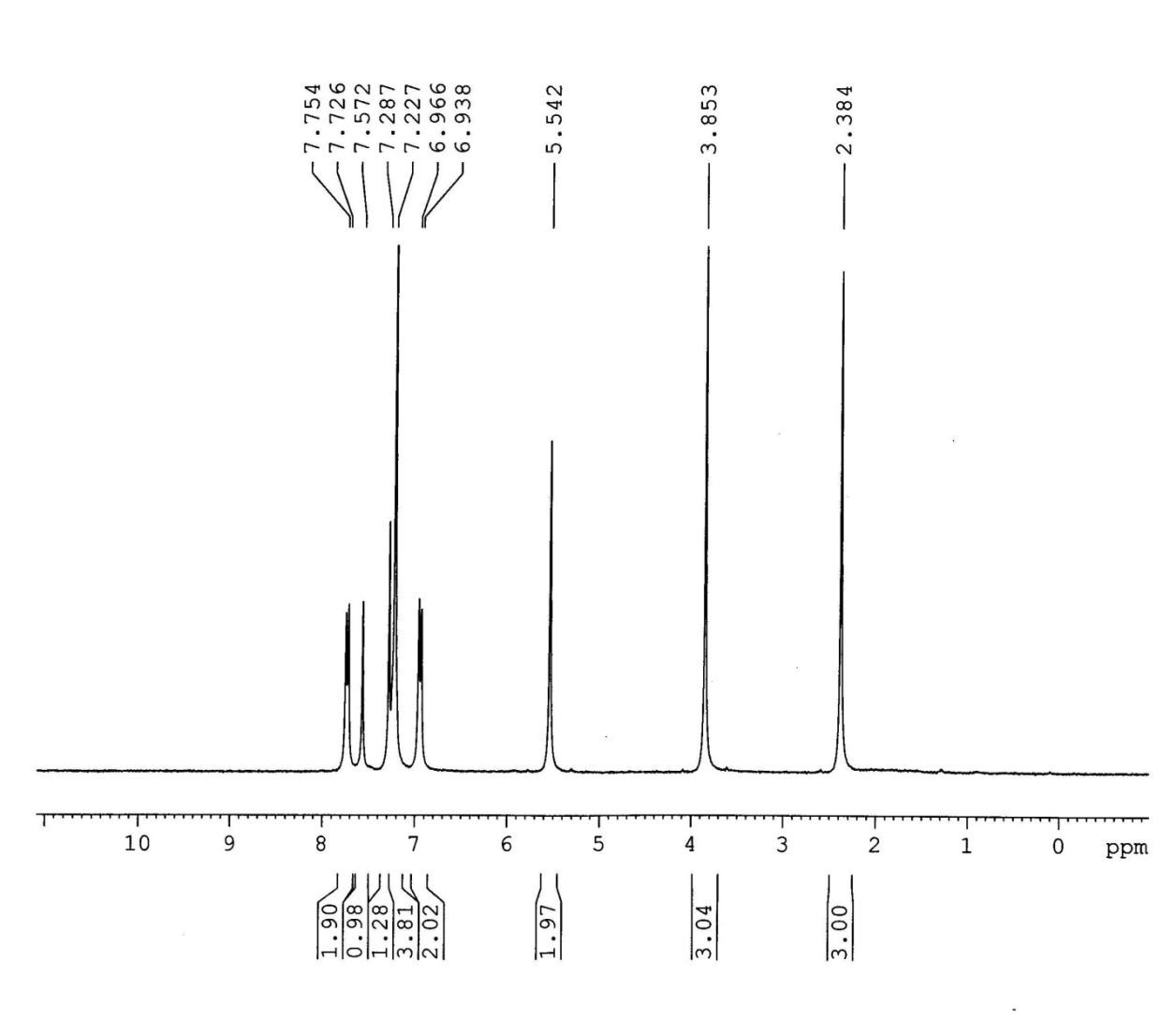 | |

**Fig. S5.**^1^H NMR of **3d** product in CDCl_3_

|  | 1-(4-bromobenzyl)-4-(*p*-tolyl)-1*H*-1,2,3-triazole (**3g**): yellow-whitepowder,mp 202- 203 ºC (2).  ^1^H NMR (300 MHz, CDCl_3_):δ=2.39 (3H,s,CH_3_), 5.55 (2H,s, CH_2_benzylic) , 7.19-7.28 (4H, m, H aromatic), 7.53-7.56 (2H, d, J=7.5Hz, H aromatic), 7.7-7.72 (2H, d, J=7.5Hz, H aromatic), 7.64 (1H, s,CHtriazole). |
| --- | --- |
| 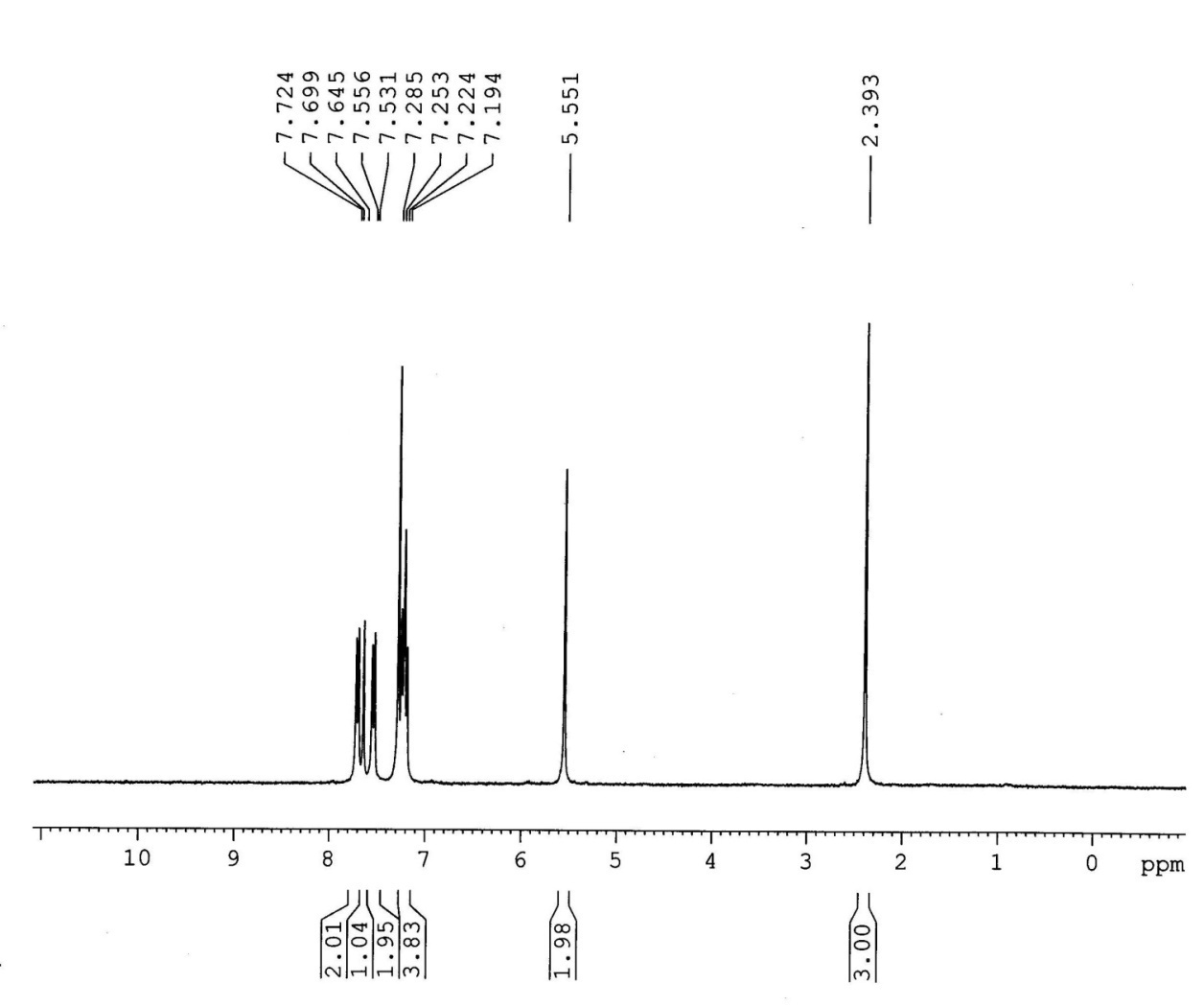 | |

**Fig. S7.**^1^H NMR of **3g** product in CDCl_3_

|  | 1-(4-methoxybenzyl)-4-(*p*-tolyl)-1*H*-1,2,3-triazole (**3m**): white powder, mp 133-136ºC(5). ^1^H NMR (300 MHz, CDCl_3_): δ=2.37 (3H,s, CH_3_), 3.83 (3H, s, OCH_3_), δ=5.51 (2H ,s, CH_2_ benzylic), 6.91-6.94 (2H, d, J=8.7 Hz, H aromatic), 7.20-7.3 (4H, m, H aromatic), 7.68-7.71 (2H, d, J=8.1 Hz, H aromatic), δ=7.6 (1H, s,CHtriazole). |
| --- | --- |
| 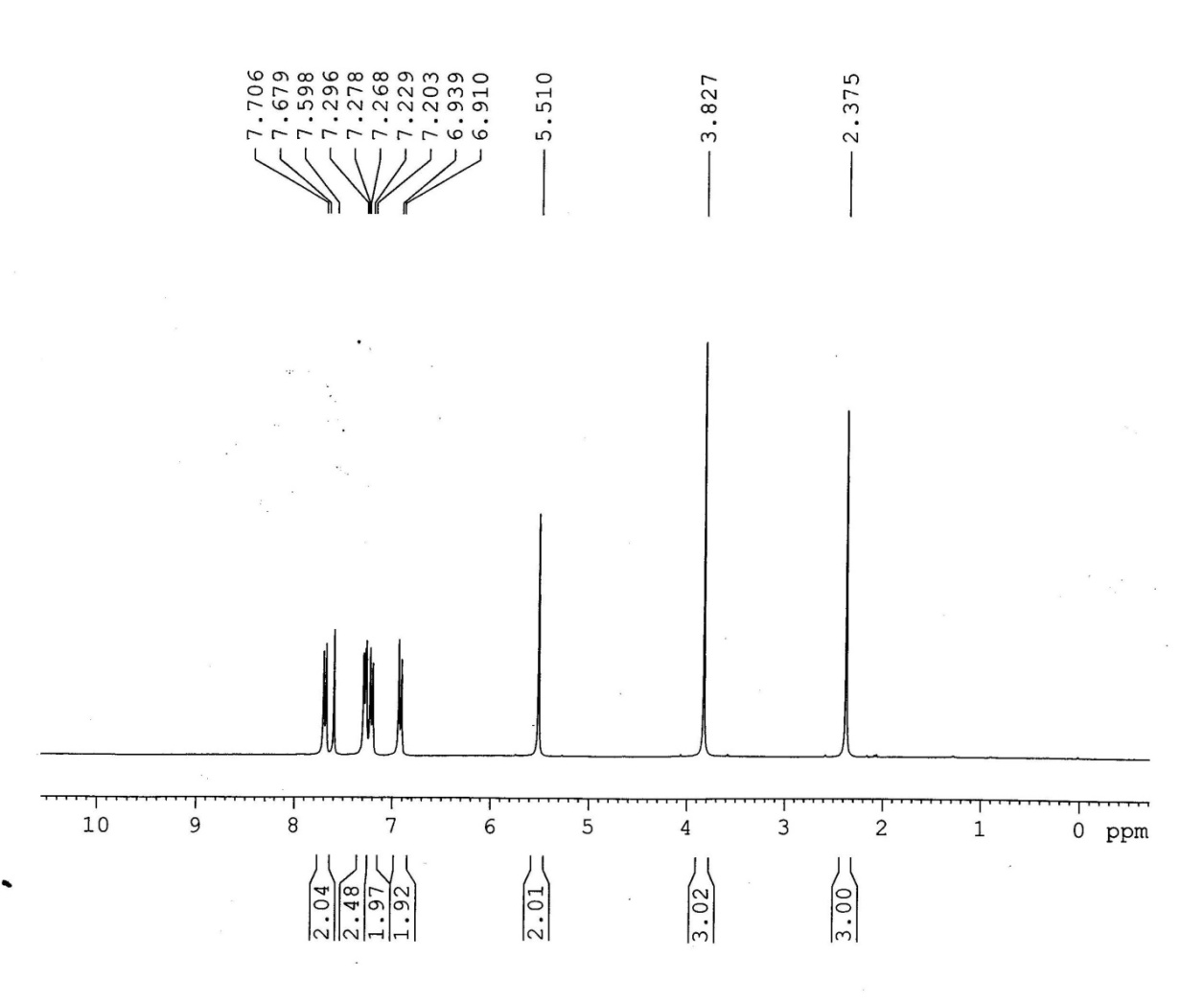 | |

**Fig. S6.**^1^H NMR of **3m** product in CDCl_3_

|  | Methyl-1-benzyl-1*H*-1,2,3-triazole-4-carboxylate (**3p**): white powder; mp 105-107ºC (6).1H NMR (300 MHz, CDCl3): δ=3.93 (s, 3H, OCH3), 5.59 (s, 2H, CH2 benzylic), 7.28-7.41 (m, 5H, H aromatic), 7.99 (s, 1H, CH triazole. |
| --- | --- |


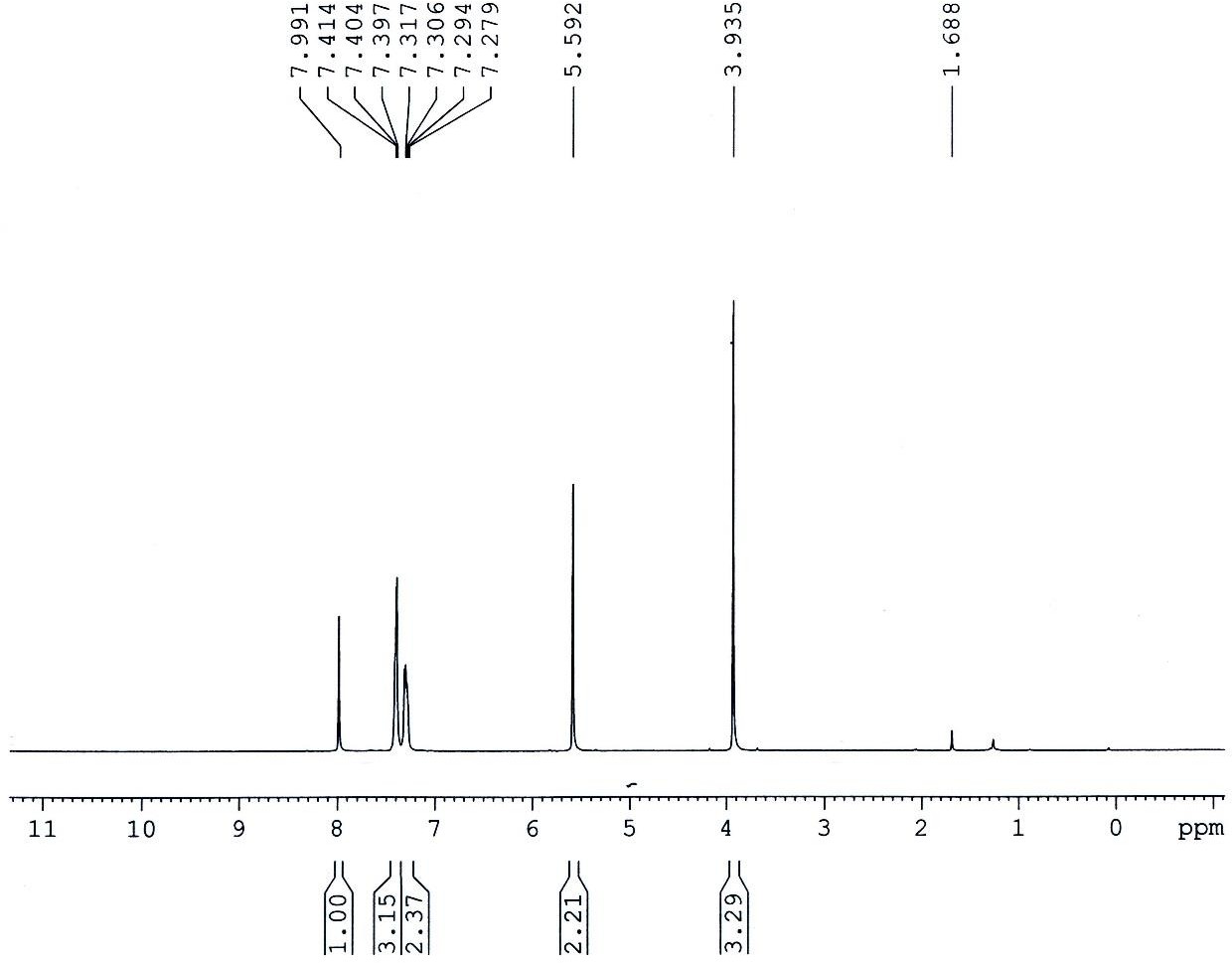


**Fig. S8.**^1^H NMR of **3p** product in CDCl_3_

|  | 1-phenyl-2-(4-phenyl-1*H*-1,2,3-triazol-1-yl)ethan-1-one (**5a**): white powder, mp 166-167 ºC (7).  ^1^H NMR (300 MHz, CDCl_3_): δ=5.94 (2H, s, CH_2_ benzylic), 7.39-8.06 (10H, m, H aromatic), 7.9 (1H, s, CH triazole). |
| --- | --- |
| 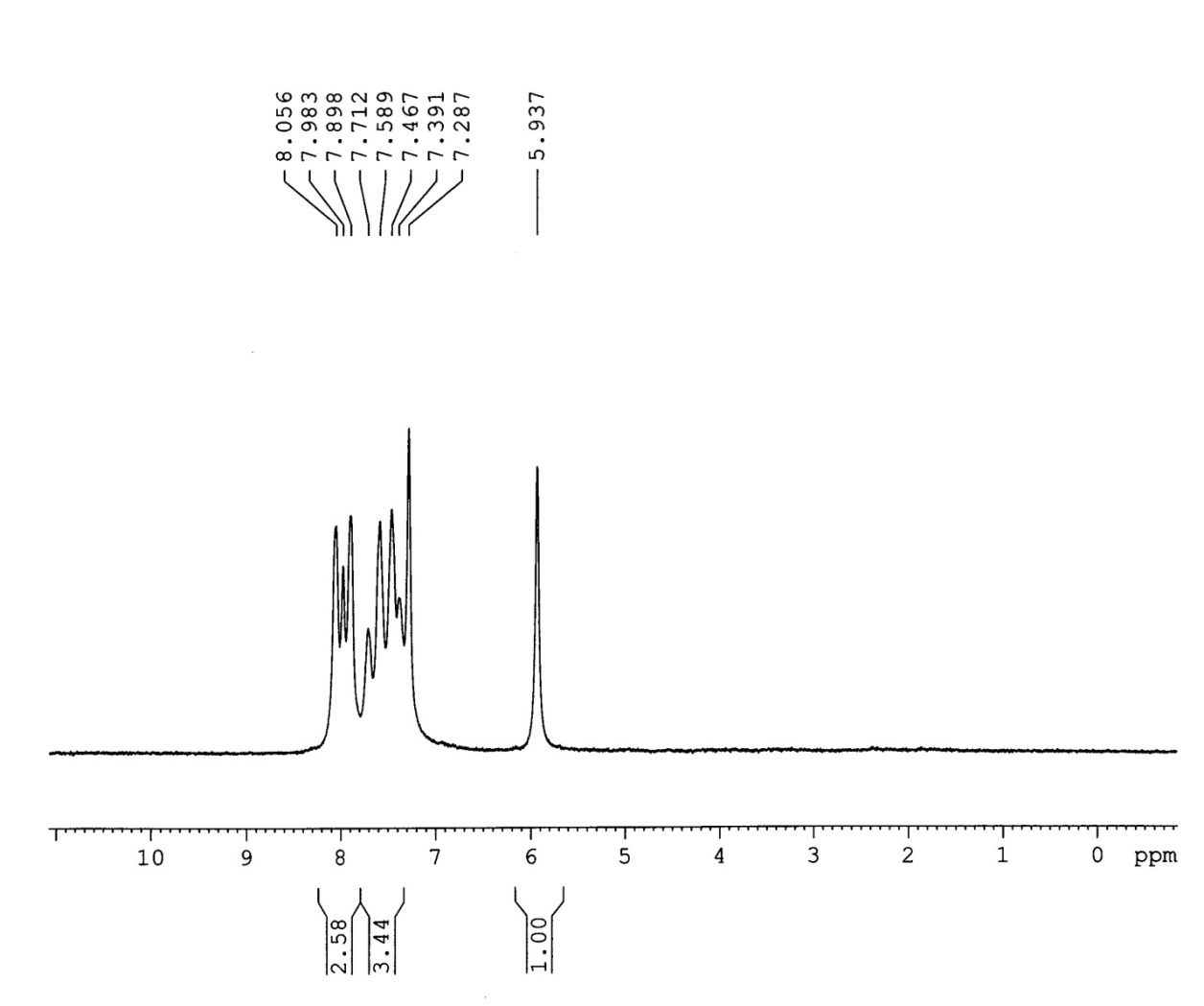 | |

**Fig. S9.**^1^H NMR of **5a** product in CDCl_3_

|  | 2-(4-(4-methoxyphenyl)-1*H*-1,2,3-triazol-1-yl)-1-phenylethan-1-one (**5b**): white powder, mp 190-191 ºC (7). ^1^H NMR (300 MHz, CDCl_3_): δ= 3.87 (3H, s, OCH3), 5.92 (2H,s,CH_2_ benzylic), 6.98-7.01(2H, d, J=7.5Hz,H aromatic), 7.55-7.84 (5H, m, H aromatic), 8.04-8.07 (2H, d , J=7.5Hz, H aromatic), 7.91 (1H, bs,CHtriazole). |
| --- | --- |
| 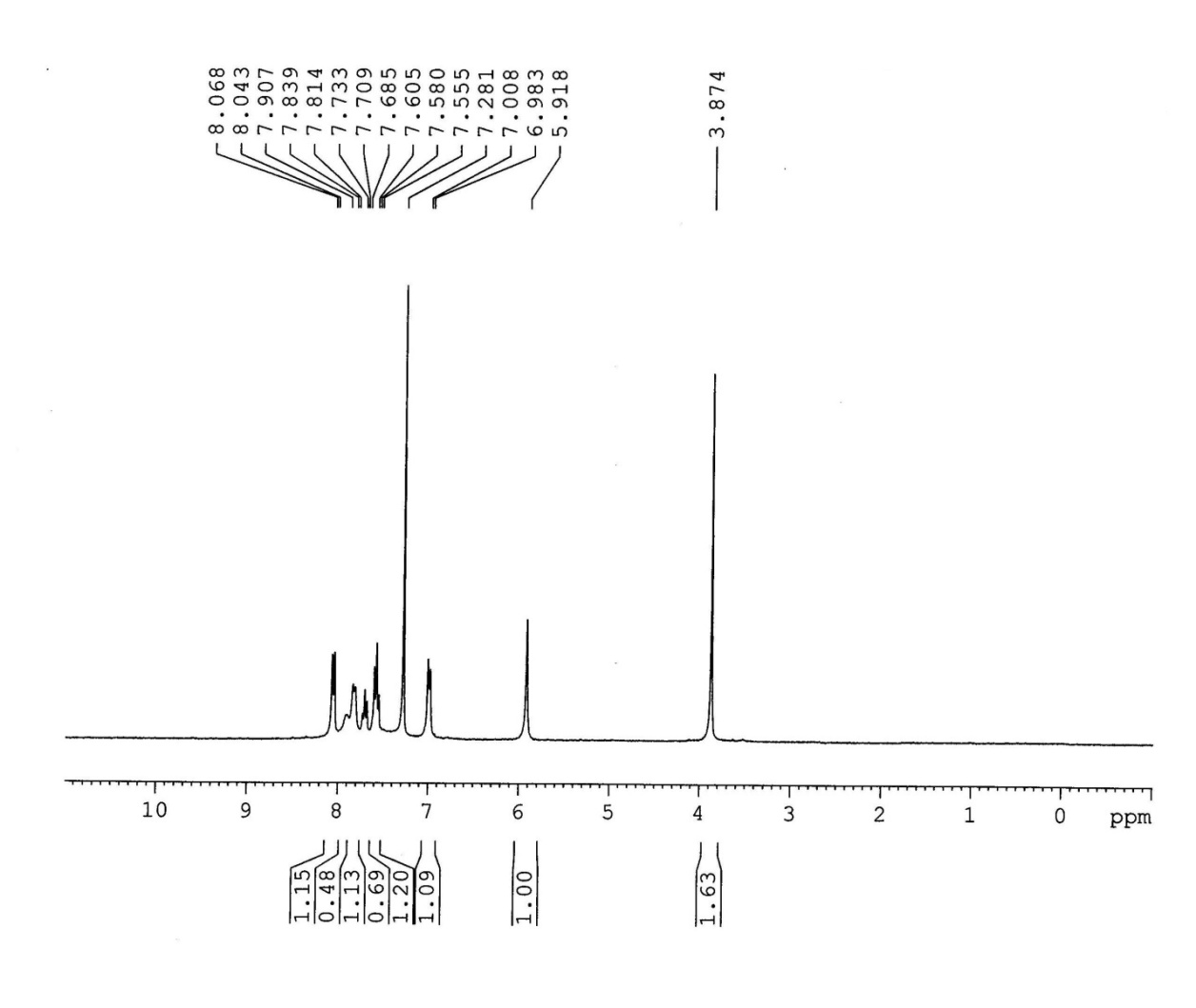 | |

**Fig. S10.**^1^H NMR of **5b** product in CDCl_3_

|  | 1-(4-chlorophenyl)-2-(4-phenyl-1*H*-1,2,3-triazole-1-yl)ethan-1-one (**5e**): white powder, mp 150-152 ºC (8).  ^1^H NMR (300 MHz, CDCl_3_): δ=5.91 (2H, s, CH_2_ benzylic), 7.29-7.58 (6H, m, H aromatic), 7.9-8.03 (4H, m, 3H aromatic, CH triazole). |
| --- | --- |
| 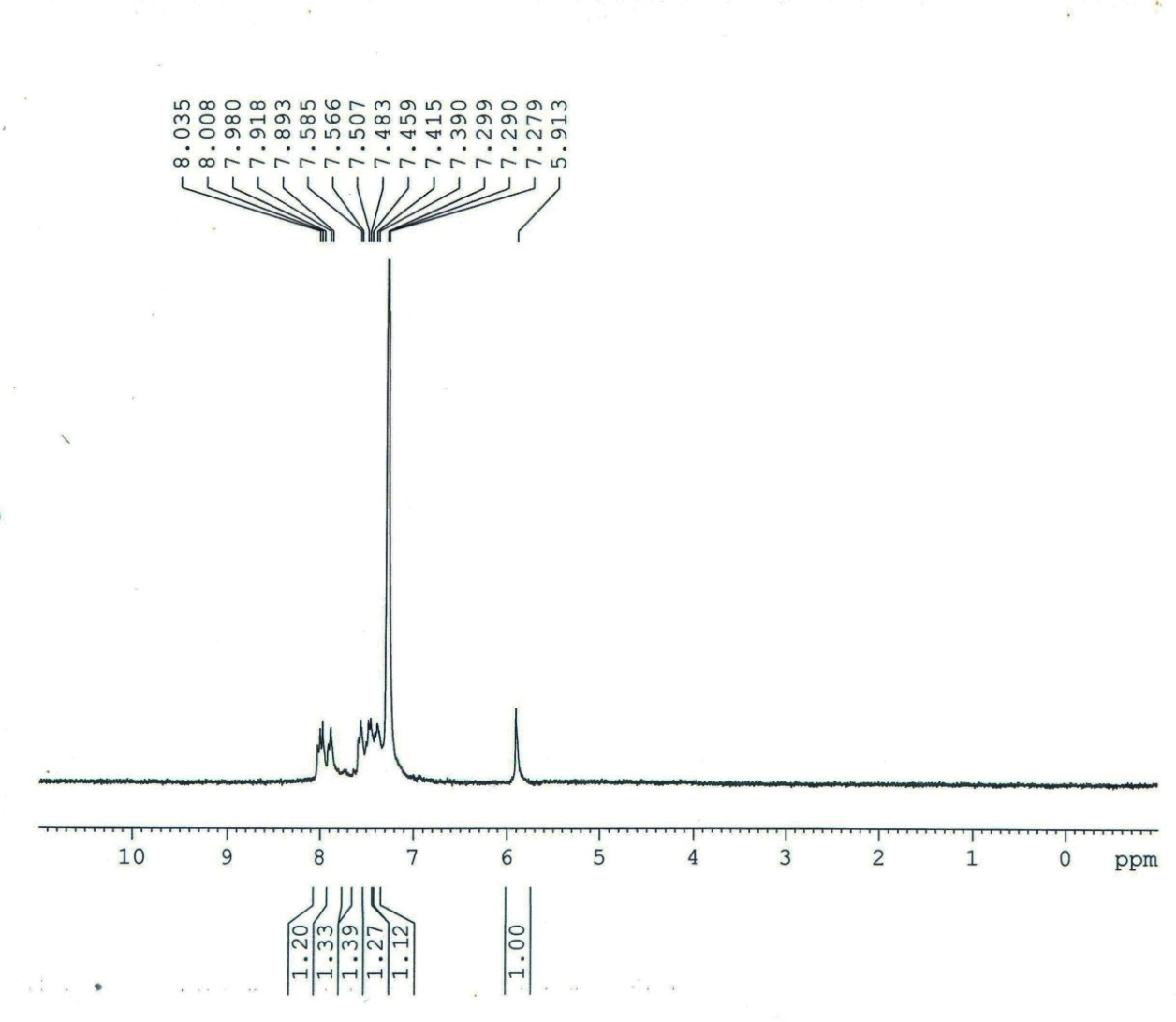 | |

**Fig. S11.**^1^H NMR of **5e** product in CDCl_3_

**References**

1. Reddy VH, Reddy YVR, Sridhar B, Reddy BVS. Green Catalytic Process for Click Synthesis Promoted by Copper Oxide Nanocomposite Supported on Graphene Oxide. Adv Synth Catal. 2016;358(7):1088-92.

2. Naeimi H, Shaabani R. Ultrasound promoted facile one pot synthesis of triazole derivatives catalyzed by functionalized graphene oxide Cu(I) complex under mild conditions. Ultrason Sonochem. 2017;34(Supplement C):246-54.

3. Asano K, Matsubara S. Effects of a Flexible Alkyl Chain on a Ligand for CuAAC Reaction. Org Lett. 2010;12(21):4988-91.

4. Szadkowska A, Staszko S, Zaorska E, Pawlowski R. A theophylline based copper N-heterocyclic carbene complex: synthesis and activity studies in green media. RSC Adv. 2016;6(50):44248-53.

5. Chavan PV, Pandit KS, Desai UV, Kulkarni MA, Wadgaonkar PP. Cellulose supported cuprous iodide nanoparticles (Cell-CuI NPs): a new heterogeneous and recyclable catalyst for the one pot synthesis of 1,4-disubstituted - 1,2,3-triazoles in water. RSC Adv. 2014;4(79):42137-46.

6. Movassagh B, Rezaei N. Polystyrene resin-supported CuI-cryptand 22 complex: A highly efficient and reusable catalyst for three-component synthesis of 1, 4-disubstituted 1, 2, 3-triazoles under aerobic conditions in water. Tetrahedron. 2014;70(46):8885-92.

7. Cha H, Lee K, Chi DY. Synthesis of N-unsubstituted 1,2,3-triazoles via aerobic oxidative N-dealkylation using copper(II) acetate. Tetrahedron. 2017;73(20):2878-85.

8. Ahmady AZ, Heidarizadeh F, Keshavarz M. Ionic Liquid Containing Copper(I): A New, Green, Homogeneous, and Reusable Catalyst for Click Cyclization. Synth Commun. 2013;43(15):2100-9.
